# Genome-wide, high-content siRNA screening identifies the Alzheimer’s genetic risk factor FERMT2 as a major modulator of APP metabolism

**DOI:** 10.1007/s00401-016-1652-z

**Published:** 2016-12-08

**Authors:** Julien Chapuis, Amandine Flaig, Benjamin Grenier-Boley, Fanny Eysert, Virginie Pottiez, Gaspard Deloison, Alexandre Vandeputte, Anne-Marie Ayral, Tiago Mendes, Shruti Desai, Alison M. Goate, John S. K. Kauwe, Florence Leroux, Adrien Herledan, Florie Demiautte, Charlotte Bauer, Fréderic Checler, Ronald C. Petersen, Kaj Blennow, Henrik Zetterberg, Lennart Minthon, Vivianna M. Van Deerlin, Virginia Man-Yee Lee, Leslie M. Shaw, John Q. Trojanowski, Marilyn Albert, Abhay Moghekar, Richard O’Brien, Elaine R. Peskind, Nicolas Malmanche, Gerard D. Schellenberg, Pierre Dourlen, Ok-Ryul Song, Carlos Cruchaga, Philippe Amouyel, Benoit Deprez, Priscille Brodin, Jean-Charles Lambert, ADGC, Alzheimer’s Disease Neuroimaging Initiative

**Affiliations:** 1 Laboratoire d’Excellence Distalz, Univ. Lille, Unité INSERM 1167, Institut Pasteur de Lille, BP 245, 1 rue du professeur Calmette, 59000 Lille cedex, France; 2 Univ. Lille, CNRS, INSERM, CHU Lille, Institut Pasteur de Lille, U1019 - UMR 8204 - CIIL - Center for Infection and Immunity of Lille, 59000 Lille, France; 3 Univ. Lille, INSERM, Institut Pasteur de Lille, U1177 - Drugs and Molecules for Living Systems, 59000 Lille, France; 4 Department of Psychiatry, Washington University School of Medicine, St. Louis, MO USA; 5 Department of Neurology, Washington University School of Medicine, St. Louis, MO USA; 6 Departments of Biology and Neuroscience, Brigham Young University, Provo, USA; 7 Laboratoire d’Excellence DistALZ, Université Côte d’Azur, INSERM, CNRS, IPMC, France, 660 route des Lucioles, Sophia-Antipolis, 06560 Valbonne, France; 8 Department of Neurology, Mayo Clinic, Rochester, MN USA; 9 Clinical Neurochemistry Laboratory, Department of Neuroscience and Physiology, Sahlgren’s University Hospital, Mölndal, Gothenburg, Sweden; 10 Department of Molecular Neuroscience, UCL Institute of Neurology, Queen Square, London, UK; 11 Clinical Memory Research Unit, Dept of Clinical Sciences, Lund University, Lund, Sweden; 12 Department of Pathology and Laboratory Medicine, Perelman School of Medicine at the University of Pennsylvania, Philadelphia, PA USA; 13 Department of Neurology, Johns Hopkins University School of Medicine, Baltimore, MD USA; 14 Department of Neurology, Duke Medical Center, Box 2900, Durham, NC 27710 USA; 15 Departments of Psychiatry and Behavioral Sciences, Veterans Affairs Northwest Network Mental Illness Research, Education, and Clinical Center, University of Washington School of Medicine, Seattle, USA; 16 Stellar-Chance Laboratories, Perelman School of Medicine, University of Pennsylvania, Philadelphia, USA

## Abstract

Genome-wide association studies (GWASs) have identified 19 susceptibility loci for Alzheimer’s disease (AD). However, understanding how these genes are involved in the pathophysiology of AD is one of the main challenges of the “post-GWAS” era. At least 123 genes are located within the 19 susceptibility loci; hence, a conventional approach (studying the genes one by one) would not be time- and cost-effective. We therefore developed a genome-wide, high-content siRNA screening approach and used it to assess the functional impact of gene under-expression on APP metabolism. We found that 832 genes modulated APP metabolism. Eight of these genes were located within AD susceptibility loci. Only FERMT2 (a β3-integrin co-activator) was also significantly associated with a variation in cerebrospinal fluid Aβ peptide levels in 2886 AD cases. Lastly, we showed that the under-expression of FERMT2 increases Aβ peptide production by raising levels of mature APP at the cell surface and facilitating its recycling. Taken as a whole, our data suggest that FERMT2 modulates the AD risk by regulating APP metabolism and Aβ peptide production.

**Electronic supplementary material:**

The online version of this article (doi:10.1007/s00401-016-1652-z) contains supplementary material, which is available to authorized users.

## Introduction

Alzheimer’s disease (AD) is a progressive, neurodegenerative disorder. It is the leading cause of dementia worldwide. Memory loss and cognitive impairments are invariant, early signs of the AD, whereas hippocampal atrophy is one of the earliest histological hallmarks of the disease. Two main lesions are found in the AD brain: the neurofibrillary tangles formed by tau aggregation in neurons, and the senile plaques formed primarily by aggregated amyloid-β (Aβ) peptides in the parenchyma. The identification of familial, AD-linked mutations in the genes for amyloid-β precursor protein (*APP*) and presenilin (*PS1* and *PS2*) associated with deregulation of Aβ peptide production suggests that APP metabolism is at the heart of the disease process. This hypothesis was recently strengthened by the discovery of a rare *APP* mutation that lowered both Aβ peptide production and the AD risk [12]. Lastly, consistent evidence also suggests that common genetic risk factors for late-onset AD (LOAD)—including the *APOE* gene—may be involved in Aβ clearance in the brain [13]. Taken as a whole, these observations indicate that Aβ production via the deregulation of APP metabolism should still be considered as a key pathogenic factor in AD.

Three main proteases (α-, β- and γ- secretases) are involved in APP processing through (1) the amyloidogenic pathway (β- and γ-secretases), leading to Aβ production, and (2) the non-amyloidogenic pathway (α- and γ -secretase), which prevents Aβ generation by cleaving APP within the Aβ sequence [6]. Recently, new δ- and η-secretase activities have been characterized [1, 26, 28], indicating that additional APP processing pathways may exist and may thus modulate Aβ loads. In addition to secretase activities, APP trafficking in the secretory pathway and APP’s fate are also essential factors in APP metabolism. APP matures in the endoplasmic reticulum and the Golgi apparatus and is then transported to the cell surface. Alternatively, APP can reach lysosomal compartments, where it undergoes proteolytic inactivation [15]. The equilibrium between these two compartments is driven by APP trafficking and maturation. *O*- and *N*-glycosylation are prerequisites for making APP available to the secretases at the cell surface and in the endosomal system, and thus for Aβ production. In this context, the various mechanisms that control APP trafficking are being intensively investigated. However, a large proportion of the key molecular players in APP trafficking have yet to been characterized.

Interestingly, new susceptibility loci for LOAD have been identified using genome-wide association studies (GWASs). One can reasonably assume that some of these genetic factors are involved in APP metabolism and Aβ production. However, it is important to bear in mind that GWAS loci may contain several genes; hence, complex linkage disequilibrium patterns with the sentinel SNP in some of these loci may make it impossible to determine which gene is responsible for the observed signal. Indeed, there are 123 genes of interest within the 19 genomic risk regions identified in the International Genomics of Alzheimer’s Project (IGAP) [9]. Even when a causative gene is eventually characterized, it is often difficult to establish a link with the pathophysiology of AD on the basis of literature data alone.

Given this context, we are seeking to develop novel, powerful approaches for empirically testing several GWAS-identified genes in cell-based or animal models. We therefore developed a genome-wide, high-content siRNA screening approach and used it to assess the functional impact of gene under-expression on APP metabolism.

## Methods

### HSC assay

**Fig. 1:**
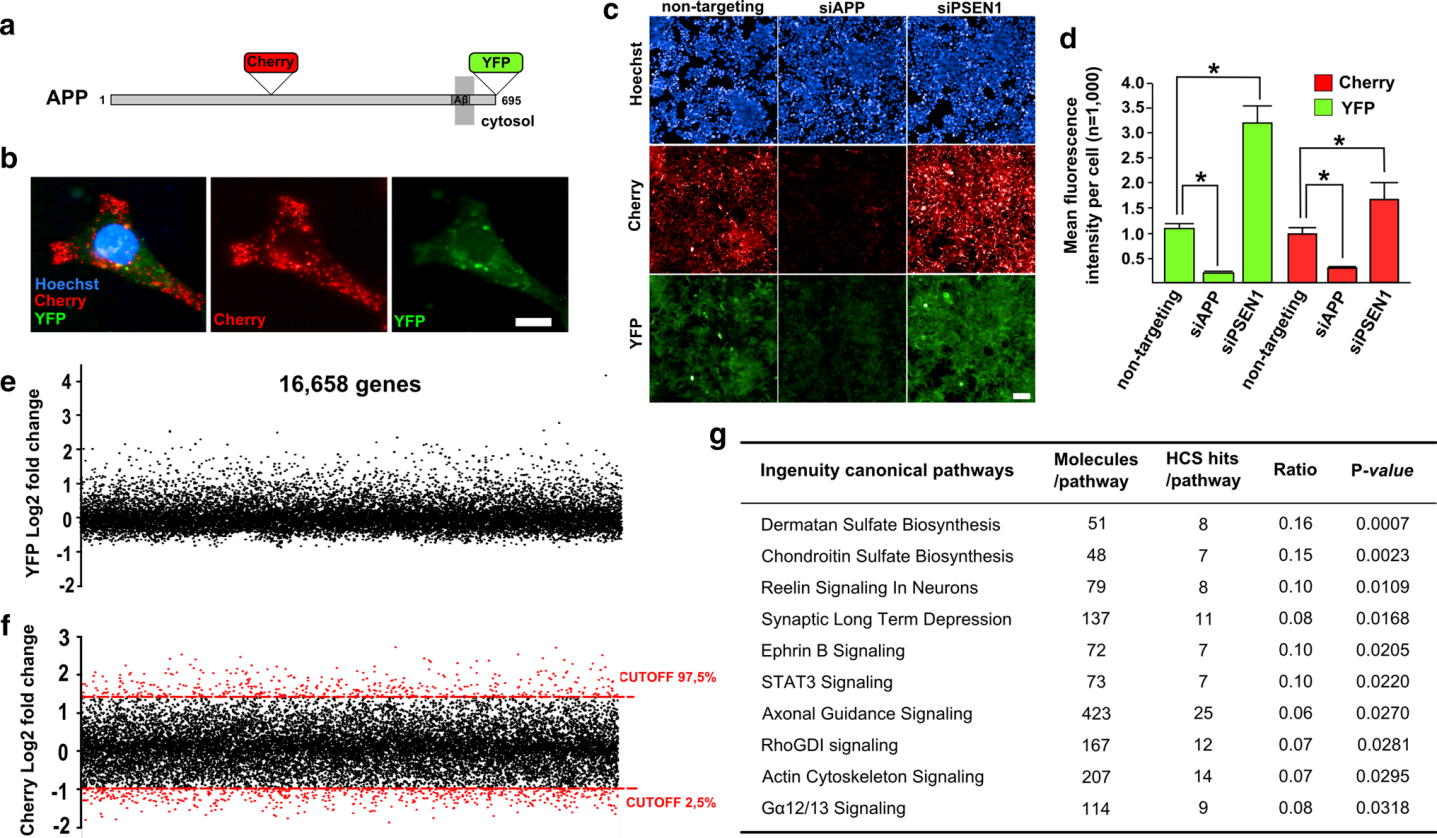
Genome-wide high-content siRNA screening identifies modulators of APP metabolism. **a** Schematic representation of APP, showing the point in the sequence at which the fluorescent proteins (cherry and YFP) were inserted. **b** Representative fluorescence microscopy images, showing HEK293 cells transfected with double-tagged APP (cherry-APP-YFP) and stained with Hoechst reagent. *Scale bar* 10 µm. **c** Representative fluorescence microscopy images showing the impact of siRNA transfection (non-targeting, siAPP or siPSEN1) on the mCherry and YFP intensities. *Scale bar* 100 µm. **d** Quantification of the relative mean fluorescence intensity of mCherry and YFP signals per cell after siRNA transfection. Histograms indicate the mean ± SD values. *p < 0.05, non-parametric test. **e** Mean fluorescence intensity variations (log2 fold-change) of the YFP signal obtained after genome-wide siRNA screening in triplicate. **f** Mean fluorescence intensity variations (log2 fold-change) of the mCherry signal obtained after genome-wide siRNA screening in triplicate. The mCherry signal was used to determine the 5% hits exhibiting the strongest variations (in *red*; 2.5% showing an upregulation and 2.5% showing a downregulation). **g** The best 10 canonical-pathways identified after pathway enrichment analysis using IPA

We developed HEK293 cells line stably over-expressing a mCherry-APP^695WT^-YFP. The modified APP^695WT^ protein is shown metabolized in the same way as APP^695WT^ [21]. Custom, automatic image processing was used to determine the cell count and the mean fluorescence intensity per cell for both mCherry and YFP signals in cytoplasm. We tested the sensitivity of this model by measuring the modulation of intracellular APP fluorescence after treatment with proteasome inhibitor, γ-secretase inhibitor or transfection of siRNAs directed against APP and PSEN1 (Supplemental Fig 1 and Fig. 1c, d). These data demonstrated that both mCherry and YFP read-outs are markers of APP levels and APP processing. With an average of 1000 cells analyzed per well, 100% of test plates (*n* = 9) were validated with a β score higher than 3 according to the HCS guideline [2] (Supplemental Fig. 1).

### Genome-wide, high-content siRNA screening

The SiGENOME SMARTpool siRNA library (targeting the 18,107 genes of the whole human genome) was screened in HEK293 cells stably over-expressing a mCherry-APP^695wt^-YFP. Briefly, 50 nl of 10 µM solutions of each siRNA were first transferred to 384-well microtiter plates using an Echo 555 liquid handler. Next, 10 µl of D-PBS containing 0.1 µl of lipofectamine™ RNAiMax were then distributed using a BioTEK EL406 Washer Dispenser. After a 30-min incubation at room temperature (enabling the transfectant complex reaction), 40 µl of HEK293-mCherry-APP^695wt^-YFP cells were distributed onto the plates using a BioTEK EL406 Washer Dispenser, in order to obtain a final density of 3000 cells per well. The microplates were incubated for 3 days at 37 °C. The cells were then incubated with 5 µg/ml of Hoechst 33,342 at 37 °C with 5% CO_2_ (v/v) for 30 min. After removal of the cell medium, 10% formalin was added to each well and plates were incubated at room temperature for 30 min for staining and cell fixation. Lastly, the cells were stored in D-PBS, and images were acquired at 405, 488 and 561 nm with an InCell Analyser 6000 high-resolution automated confocal microscope. One field per well was read from the B1 well to the O24 well in a horizontal, serpentine acquisition mode with a 20× objective.

### HCS quantification and analysis

Customized image analysis software (Columbus 2.7, PerkinElmer) was used for the image analysis and the quantification. Hoechst staining was used for the segmentation of both nuclei and cell. Next, the mean fluorescence intensity of each mCherry and YFP signals in the cytoplasm were quantified. The mean fluorescence intensity of each signals were then normalized to the fold-change based on the non-targeting siRNA in the same plate. To evaluate the impact of each siRNA, an average of 1000 cells was analyzed per run (*n* = 3). For the quality control of HTS, we used strictly standardized mean difference with more than 3 of beta-score of two positive control (siRNA-APP and siRNA-PS1, *n* = 14 each per plate) (Supplemental Fig. 1).

### Pathway analysis

Ingenuity Pathway Analysis (IPA; Ingenuity Systems/Qiagen, Redwood City, USA) was used to map lists of significant genes to gene ontology groups and biological pathways. An Ingenuity ‘core analysis’ based on the Ingenuity Pathway Knowledge Base (gene only) was performed (2015 release) considering only molecules and/or relationships experimentally observed (direct and indirect relationships) in human (stringent filter).

### CSF biomarker datasets

CSF samples were obtained from the Knight-ADRC (*N* = 893), ADNI (*N* = 394, the Biomarkers for Older Controls at Risk for Dementia (BIOCARD) (*N* = 182), Mayo Clinic (*N* = 433), Lund University (Swedish) (*N* = 293), University of Pennsylvania (Penn) (*N* = 164), University of Washington (*N* = 375).

Cases were diagnosed with dementia of the Alzheimer’s type (DAT) according to the NINCDS-ADRDA. Control individuals were evaluated using the same criteria and showed no symptoms of cognitive impairment. All participants provided written informed consent and the ethics committee approved the informed consent procedure (IRB ID #: 201105364). 787 additional samples with biomarker data used in the analyses were obtained from the ADNI database (adni.loni.usc.edu). CSF in all studies was collected in a standardized manner. Briefly, CSF (20–35 ml) was collected at 8:00 AM after overnight fasting, as described previously [10, 11]. LPs (L4/L5) were performed by a trained neurologist using a 22-gauge Sprotte spinal needle. Samples were gently inverted to avoid gradient effects, briefly centrifuged at low speed to pellet any cellular elements, and aliquoted (500 μl) into polypropylene tubes before freezing at −84 °C. Biomarker measurements within each study were conducted using internal standards and controls to achieve consistency and reliability. However, differences in the measured values between studies were observed which are likely due to differences in the antibodies and technologies used for quantification (standard ELISA with Innotest for Knight-ADRC, UW, Swedish, German, and Mayo versus Luminex with AlzBio3 for ADNI-1, ADNI-2, BIOCARD and Penn), ascertainment and/or handling of the CSF after collection. CSF Aβ42 and ptau181 values were log transformed in order to approximate a normal distribution. Because the CSF biomarker values were measured using two different platforms (standard ELISA with Innotest and Luminex with AlzBio3), we did not combine the raw data. For the combined analyses, we standardized the mean of the log-transformed values from each dataset to zero. No significant differences in the transformed and standardized CSF values were found between cohorts. We also performed meta-analyses for the most significant SNPs by combining the P values for each independent dataset using METAL. No major differences were found between the joint-analyses and the meta-analyses.

### Cell culture, transfections, and Western blotting (WB)

HEK293 cell lines were maintained in 1:1 DMEM F12/Opti-MEM supplemented with 10% fetal bovine serum, penicillin, and streptomycin at 37 °C in a humidified atmosphere with 5% CO_2_. Prior to transfection, cells were plated at a density of ~70%. transient transfection of FERMT2 cDNA (cloned into a pcDNA4 vector; GeneArt) was performed using Fugene HD (Invitrogen) according to the manufacturer’s instructions. For WB, cells were washed with PBS and solubilized in ice-cold lysis buffer (Tris 1 M pH 7.4; NaCl 1.5 M; Nonidet P-40 0.1%; SDS 10%; sodium orthovanadate 100 mM; sodium deoxycholate 0.5%; 1× complete protease inhibitor mixture, Roche Applied Sciences). Cell extracts (5–20 μg) were analyzed using SDS-PAGE and the antibodies listed. For WB and immunofluorescence analysis, the following antibodies were used: hFERMT2 (GTX84507, GeneTex), amyloid precursor protein C-Terminal (A8717, Sigma), actin (A2066, Sigma), β-amyloid 6E10 (SIG-39320, Biolegends), ATPase (Na^+^–K^+^) alpha subunit (a5, DSHB), Rab4 (PA3-912, Thermo Scientific Pierce), Alzheimer precursor protein A4 clone 22C11 (MAB348, Millipore). For siRNA transfection, we used Dharmacon siRNA, non-targeting (D0018100105) and siFermt2 (J01275305, J01275306, J01276307, J01275308 and L01275300). Secreted Aβ and sAPP fragments were analyzed with an AlphaLISA, as described previously [15].

### Primary neuronal cultures

Primary mixed cortical and hippocampal neuronal cultures were obtained from P0 rats, according to previously described procedures [16]. Briefly, hippocampi and cortices were isolated from newborn rats, and neurons were dissociated by trypsin digestion. Neurons were plated on poly-l-lysine-coated coverslips or six-well plates, and were incubated with minimal essential medium (MEM) supplemented with 10% fetal bovine serum, Glutamax, MEM vitamins and penicillin/streptomycin (Life Technologies), according to the manufacturer’s instructions. After 24 h, neurons were transferred into serum-free Neurobasal-A medium supplemented with B27 (Gibco, Life Technologies), Glutamax and uridine-deoxyfluorouridine for 14 days of in vitro culture. Lentivirus transductions were performed (MOI = 4) used Mission shRNA vectors (Sigma), non-targeting (05191520MN) and shFermt2 (TRCN0000191859).

### Immunofluorescence

The immunofluorescence procedure has been described previously [5]). In brief, cells were washed with PBS, fixed in PBS containing 4% paraformaldehyde for 20 min at room temperature, and then permeabilized with 0.25% (v/v) Triton X-100 in PBS for 10 min. After blocking in 1% (w/v) bovine serum albumin (BSA), cells were incubated for 2 h at room temperature with primary antibodies diluted 1/100 in PBS 1% BSA. The cells were then washed 3 times with PBS. Appropriate secondary antibodies (diluted 1/400) were applied. After washing, coverslips were mounted on slides.

For the APP internalization assay, cells were incubated with 6E10 antibody for 1 h at 4 °C in ice-cold Dulbecco’s modified Eagle’s medium supplemented with 1% (w/v) bovine serum albumin and then washed and incubated at 37 °C for the times indicated. Cells were fixed as described above.

### Cell surface biotinylation

HEK293-APP695^WT^ cells or primary neuronal cultures were transfected in 100 mm dishes. After 48 h of transfection, cell surface proteins were biotinylated using sulfo-NHS-SS-biotin, as per the supplier’s recommendations (Cell Surface Protein Isolation Kit, Pierce). Briefly, cells were incubated with cold PBS containing sulfo-NHS-SS-biotin for 30 min at 4 °C, with gentle rocking. Cells were then lysed and immunoprecipitated with streptavidin beads. Precipitated proteins were eluted from the avidin beads with loading buffer containing 50 mM DTT, heated for 5 min at 95 °C, and analyzed by WB.

### Statistical analysis

The robustness of the replicates between the 3 screens was assessed using the standard deviation (SD). In average, the SD was of 11.5% (±7.3%) for the 16,658 genes and 12.4% (±8.1%) for the 832 hits. Only 661 genes exhibited experiments with an SD superior of 25% for the 16,658 genes (54 in the 832 list). Associations between the CSF Aβ42 level and the genetic variants were analyzed as previously reported [7]. Our analysis included a total of 5,815,690 imputed and genotyped variants. We used Plink to analyze the SNPs’ associations with CSF biomarker levels. Age, gender, site, and the three principal component factors for the population structure were included as covariates.

## Results

### Systematic high-content screening for genes that modulate APP metabolism

To identify modulators of APP metabolism, we developed a cell-based, high-content assay for the rapid detection and quantification of intracellular APP fragments in HEK293 cells stably over-expressing a mCherry-APP^695WT^-YFP (Fig. 1a, b, Supplemental Fig. 1). After customization for automatic image processing, we screened a genome-wide bank of 18,107 human siRNAs (SMARTpool) by analyzing the impact of transfection in our HEK293 model (in a 384-well plate format). For quality control procedures, the siRNA-PSEN1 and siRNA-APP (Fig. 1c, d) were used to calculate the strictly standardized mean difference (denoted as β in Supplemental Fig. 1). The complete screen was performed in triplicate, and only plates with *β* > 3 were analyzed (98.5%) as recommended in high-content screening (HCS) guidelines [2]. This procedure led us to select 17,354 siRNAs. Furthermore, experiments in which less than 300 cells per well were analyzable were excluded (mean ± standard deviation cell count per well for the whole HCS experiment: 795 ± 345). The impact of 16,653 siRNA transfections on APP metabolism was then assessed, and the mean variation in both mCherry and YFP signals was normalized against the fluorescence intensity of non-targeting siRNA. It is noteworthy that transfection of siRNA targeting genes already known to modulate APP metabolism (*ADAM10*, *PSEN1*, *BACE1* and *SORL1*) showed significant variations for mCherry and YFP signals, compared with non-targeting siRNA (Supplemental Fig. 2).

Because YFP fluorescence is detected weakly, due to its rapid turnover at the membrane [21], it limited the detection of a down-variation for this signal (Fig. 1e). Thus, we decided to focus on the mCherry signal as the main read-out for selecting the 5% of hits showing the strongest variations (2.5% upregulated and 2.5% down-regulated) (Fig. 1f). In all, 832 hits with a potential impact on APP metabolism were selected (Fig. 1f; Supplemental Table 1). In average, the standard deviation was of 12.4% (±8.1%) for the 832 hits.

Starting from this list of genes, we investigated potential protein–protein interaction networks by using Ingenuity Pathway Analysis (IPA) software (http://www.ingenuity.com/products/ipa). This hypothesis-free approach described a complex interactive network that was primarily centered on APP (Supplemental Fig. 3). APP was the protein with the highest number of interactions with other proteins in the network—indicating that our HCS approach efficiently detected proteins known to interact with APP. Gene enrichment analysis (using IPA) of the 832 hits identified 10 significantly over-represented pathways; notably, dermatan and chondroitin sulfate biosynthesis (*p* = 0.0007) is involved in the composition of the extracellular matrix and the glycosylation of proteins like APP (Fig. 1g). All the other pathways were signaling pathways mainly involving integrin, paxillin or receptor tyrosine kinase signaling (Supplemental Fig. 4). These pathways (axogenesis; neuron and neurite development; cell–cell junction maintenance; cell morphogenesis and projection; and integrin-mediated signaling) have been already implicated in the modulation of Aβ secretion [3].

### Identification of FERMT2 as a genetic risk factor that modulates APP metabolism

**Fig. 2:**
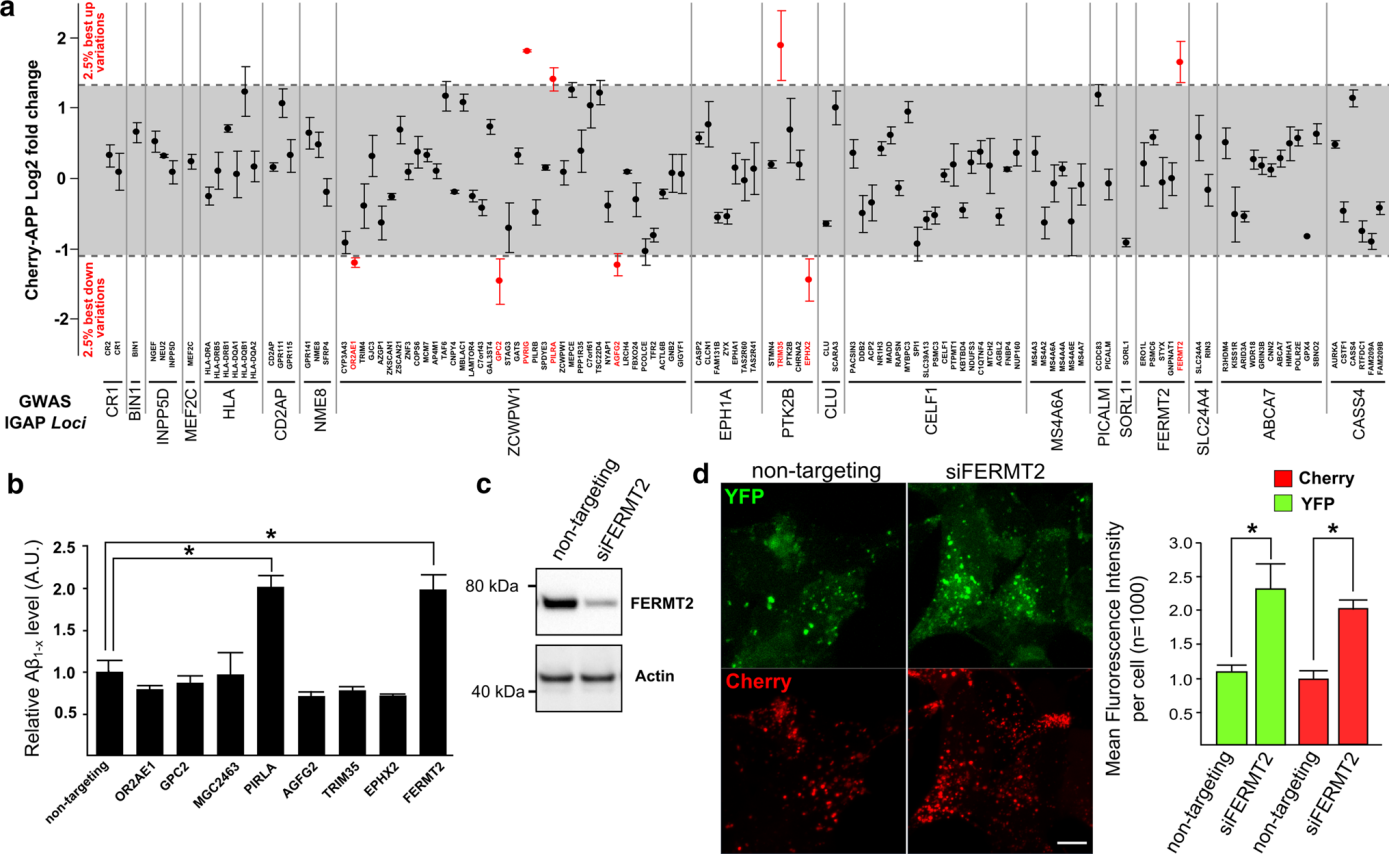
Cross-correlations between HCS and GWAS data. **a** Mean variations in mCherry fluorescence intensity after the silencing of genes associated with the AD risk in the IGAP’s meta-analysis. Eight genes (in *red*) were included in the best 5% variations, based on the HCS data. **b** Impact of the silencing of the 8 hits on the Aβ_1-X_ secretion level in the medium of the HEK293-mCherry-APP-YFP cell line. Histograms indicate mean ± SD. *p < 0.05, non-parametric test. **c** Validation of FERMT2 silencing after transfection with the siRNA-FERMT2 SMARTpool used in HCS. **d** Representative fluorescence microscopy images and quantification showing the impact of FERMT2 silencing on mCherry and YFP intensity based on HCS data. *Scale bar* 10 µm

We used HCS data to investigate the function of the genes within the LOAD risk loci recently reported in the IGAP’s meta-analysis of GWASs [14]. Of the 123 known genes reaching genome-wide significance, 8 were included in the top 5% of hits: *OR2AE1*, *GPC2*, *PVRIG*, *PILRA*, *AGFG2*, *TRIM35*, *EPHX2* and *FERMT2* (Fig. 2a) suggesting that these genes could be involved in the AD process via the regulation of APP metabolism. However, only PIRLA and FERMT2 showed an effect on the Aβ_1-x_ secretion in HEK293-mCherry-APP^695wt^-YFP (Fig. 2b). Lastly, to assess the potential impact of these genes on Aβ peptide levels in humans, we measured the association between SNPs in these 8 genes and cerebrospinal fluid (CSF) Aβ42 peptide levels in a large sample (*n* = 2886) of AD cases [7]. After gene-wide correction (Bonferroni, *p* < 0.006), we found that only SNPs within *FERMT2* were associated with low Aβ42 peptide levels (*p* = 0.0006; Table 1).

**Table 1:** The associations between the eight genes located in IGAP loci and the CSF Aβ42 concentration (*n* = 2886 AD cases)

Modulators of APP metabolism in IGAP loci							Association with the CSF Ab42 level*		
Chr.	IGAP locus	Gene	GFP log2 fold-change		mCherry log2 fold-change		SNP	*β* score	*p* value
7	*ZCWPW1*	*OR2AE1*	−0.58	±0.05	−1.18	±0.07	rs35649099	−0.01422	0.2026
		*GPC2*	−0.68	±0.09	−1.45	±0.32	rs12705074	−0.01702	0.0384
		*MGC2463*	1.29	±0.04	1.83	±0.02	rs150436753	0.01965	0.1215
		*PILRA*	1.17	±0.10	1.43	±0.16	rs28714213	0.01737	0.0171
		*AGFG2*	−0.34	±0.05	−1.21	±0.16	rs78951820	−0.03433	0.0247
8	*PTK2B*	*TRIM35*	1.84	±0.30	1.91	±0.50	rs77389621	−0.02716	0.0584
		*EPHX2*	−0.46	±0.08	−1.42	±0.30	rs7341557	0.02001	0.0160
**14**	***FERMT2***	***FERMT2***	**1.19**	±**0.34**	**1.67**	±**0.28**	**rs62003531**	−**0.02745**	**0.0006**

Bold represents significance after correction for multiple testing

* Linear regression, adjusted for age and gender

Of note, according to the RNA-Seq transcriptome and splicing database ( http://web.stanford.edu/group/barres_lab/brain_rnaseq.html), FERMT2 (but not PIRLA) is expressed in neurons which are the main sources of Aβ in the brain. Similar results were observed in another database focusing on hippocampal neurons (http://hipposeq.janelia.org/; data not shown).

All together, these observations highlighted the potential role of *FERMT2* in the AD process via the modulation of APP metabolism and Aβ peptide generation. We first validated the silencing of FERMT2 after transfection of the SMARTpool siRNA library used for HCS (Fig. 2c). FERMT2 knock-down was associated with the accumulation of both mCherry and YFP signals (Fig. 2d). Thus, we decided to focus on characterizing the impact of FERMT2 on APP metabolism in cell-based models.

### FERMT2 modulates APP processing and metabolite secretion

To rule out a potential off-target effect of the siRNA-FERMT2 SMARTpool used for HCS (four different siRNAs), we first validated the mCherry and YFP signal variations associated with FERMT2 silencing by assessing the effect of each siRNA-FERMT2 independently (*n* = 4). Three siRNA-FERMT2 were associated with an increase in both mCherry and YFP signals, which was consistent with the effect observed when using the siRNA pool (Supplemental Fig. 5).

**Fig. 3:**
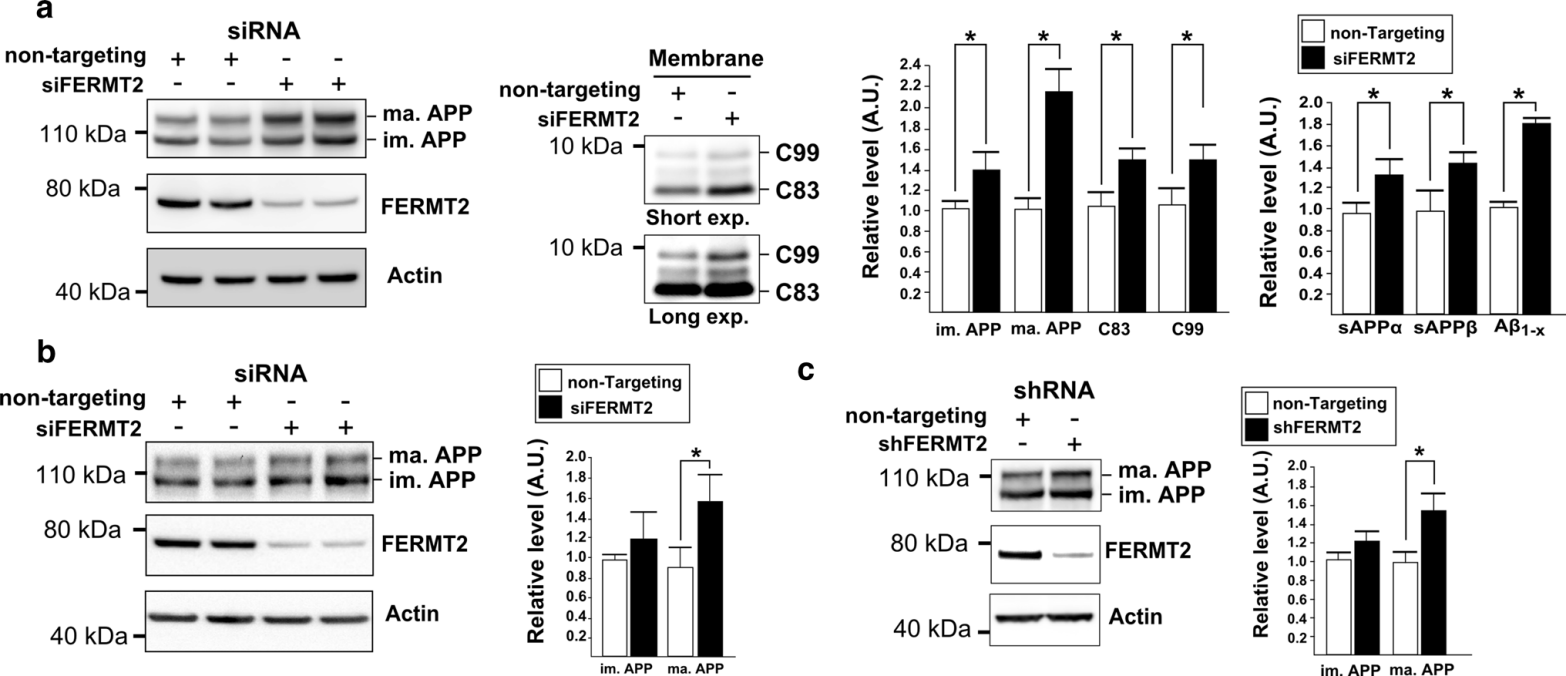
Characterization of the impact of FERMT2 on APP metabolism. **a** Impact of FERMT2 silencing on APP metabolism in the HEK293-APP^695WT^ cell line. Cells transiently transfected with siFERMT2 or non-targeting siRNA were analyzed by WB using anti-APP C-terminal, anti-FERMT2 or anti-actin antibodies. sAPPα, sAPPβ and Aβ_1-X_ secreted into conditioned medium were assayed using an AlphaLISA. ma. APP, mature APP; im. APP, immature APP. Densitometric analyses and WB quantifications from three independent experiments are shown. Histograms indicate the mean ± SD. a.u., arbitrary units. *p < 0.05, non-parametric test. **b** Impact of FERMT2 silencing on the mature APP levels in HEK293 cells endogenously expressing APP. **c** Quantification of mature and immature APP levels after lentiviral transduction with shRNA against FERMT2 in a primary neuronal culture endogenously expressing APP

To further evaluate the impact of FERMT2 silencing on APP metabolism, we quantified mature and immature forms of APP and the various metabolites of APP. In the HEK293 cell line stably over-expressing APP^695WT^ (HEK293-APP^695WT^), we observed that a strong increase in mature APP levels was associated with the accumulation of all the APP-derived substrates for α-, β- and γ-secretases (C83 and C99 intracellular C-terminal fragments of APP produced, respectively, by α- and β-secretases, as well as APPα, sAPPβ and Aβ secretions) (Fig. 3a). Again, similar results were obtained using different siRNA-FERMT2 sequences (Supplemental Fig. 6).

Next, we assessed the impact of FERMT2 silencing on endogenous APP metabolism in HEK293. As observed in HEK293-APP^695WT^ cells, FERMT2 silencing resulted in a significant increase in mature APP levels (Fig. 3b). It is noteworthy that mature APP levels also rose after transfection of siRNA-FERMT2 into the HEK293-mCherry-APP^695wt^-YFP cell line (Supplemental Fig. 7)—showing that the mechanisms were consistent in our cell models expressing APP^695WT^ in the presence or absence of mCherry and YFP tags.

Lastly, we confirmed the increase in neuron levels of mature APP after lentiviral transduction with shRNA against FERMT2 in a primary neuronal culture (PNC) endogenously expressing APP (Fig. 3c). Taken as a whole, these results show that FREMT2 silencing (using either siRNA or shRNA) controls the mature APP levels in various models overexpressing (or not) APP.

### FERMT2 expression controls cell surface levels of APP

**Fig. 4:**
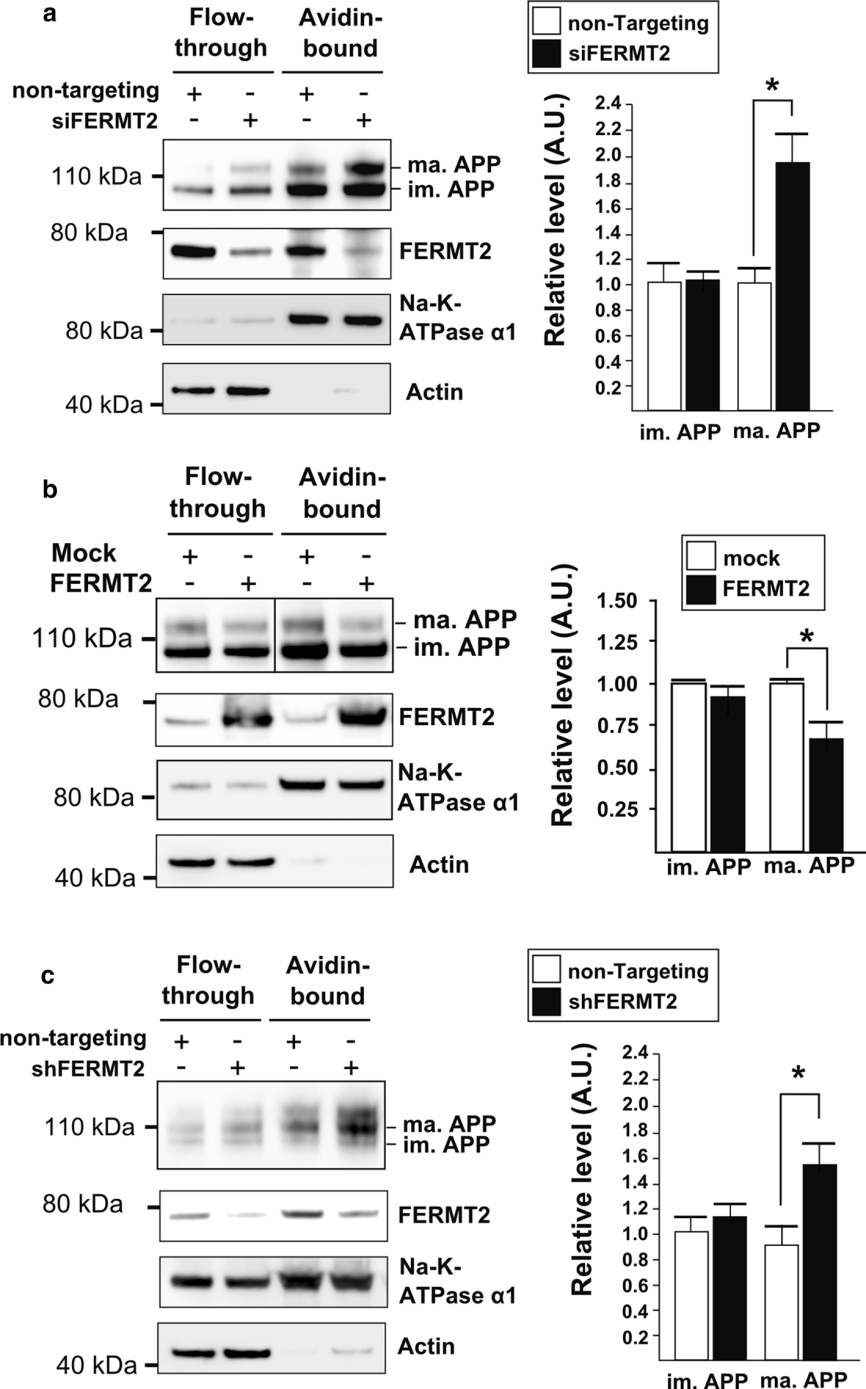
FERMT2 expression controls the cell surface level of mature APP. **a** Cell-surface-biotinylated proteins from HEK293-APP^695WT^ cells transiently transfected with siFERMT2 or non-targeting siRNA. Cell extracts were precipitated with immobilized avidin and analyzed by WB using antibodies against APP, FERMT2, actin (an intracellular marker), and Na–K-ATPase α1 (a cell surface marker). ma. APP, mature APP; im. APP, immature APP. Densitometric analyses and WB quantifications from three independent experiments are shown. Histograms indicate the mean ± SD. a.u., arbitrary units. *p < 0.05, non-parametric test. **b** Cell-surface-biotinylated proteins from HEK293-APP^695WT^ cells transiently transfected with FERMT2 cDNA or empty vector (Mock). **c** Cell-surface-biotinylated proteins from primary neuronal culture after lentiviral transduction with shRNA against FERMT2 or non-targeting shRNA

A large body of evidence suggests that APP is mainly cleaved by secretases at or near the plasma membrane. Since FERMT2 silencing reportedly increases the amount of CD39 and CD73 at the cell surface [18], we looked at whether FERMT2 can interfere with mature APP levels by promoting APP trafficking to the cell surface. If so, this would be consistent with our previous observation in which FERMT2 silencing appeared to be associated with a general increase in mature APP levels, but did not change immature APP levels in total cell extracts. We addressed this hypothesis more specifically by performing extracellular biotinylation experiments; these revealed that FERMT2 silencing strongly increased mature APP levels at the cell surface in HEK293-APP^695wt^ cells (Fig. 4a). Conversely, the over-expression of FERMT2 was associated with low mature APP levels at the cell surface (Fig. 4b). This observation was validated in HEK293 and PNCs (both of which endogenously expressed APP): FERMT2 silencing was systematically associated with more abundant mature APP in cell-surface-biotinylated fractions (Fig. S7 and Fig. 4c, respectively).

### FERMT2 silencing inhibits APP degradation and promotes APP recycling at the plasma membrane

**Fig. 5:**
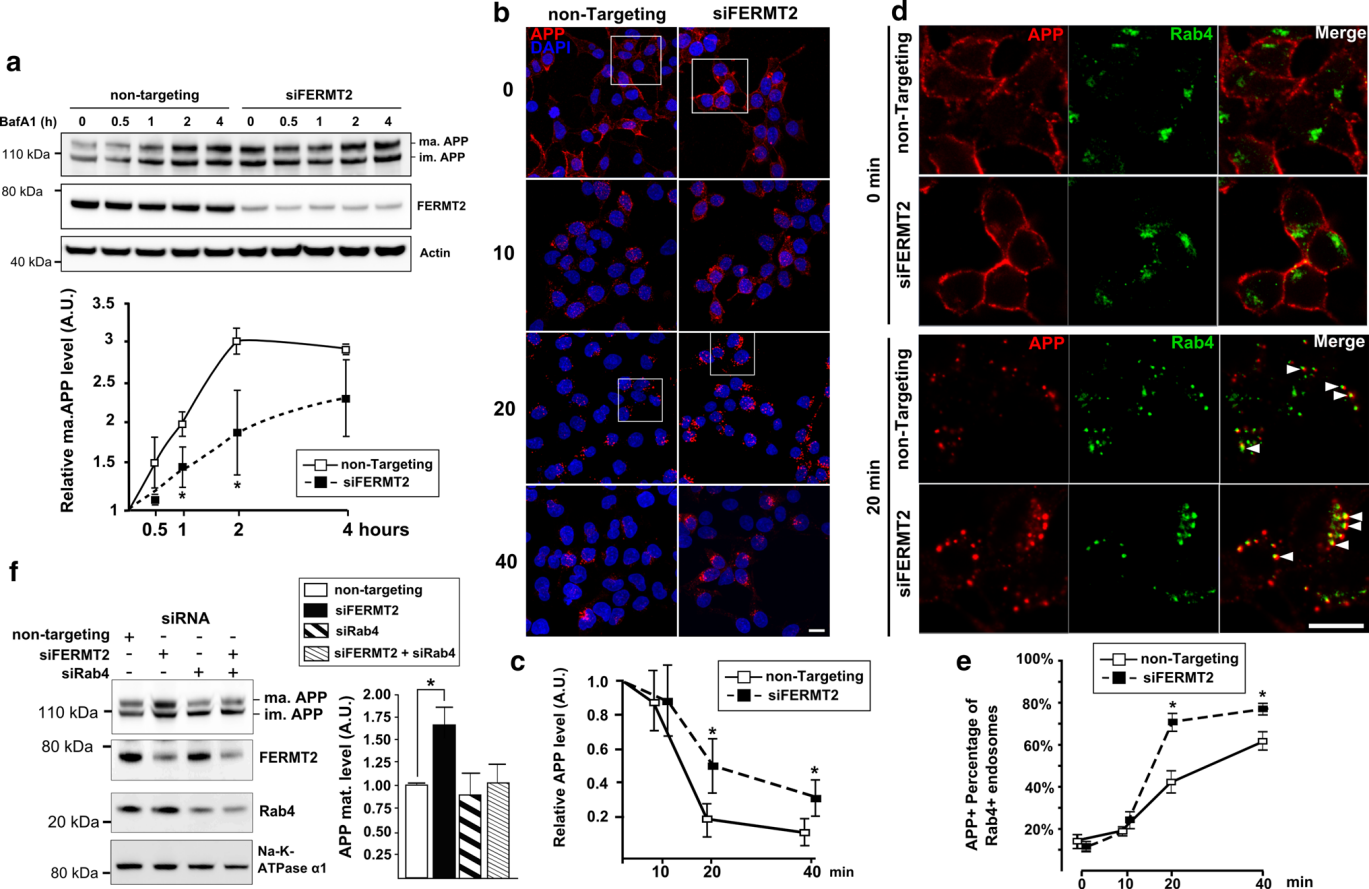
FERMT2 silencing inhibits APP degradation and promotes APP recycling at the plasma membrane. **a** HEK293-APP^695WT^ cells transiently transfected with siFERMT2 or non-targeting siRNA were treated with bafilomycin A1 (BafA1, 50 nM) for the indicated times. Cell extracts were then analyzed by WB. Densitometric analyses and mature APP levels for three independent experiments are shown. Graphs indicate the mean ± SD. *p < 0.05, non-parametric test. **b** The time course of APP endocytosis and degradation was indirectly visualized by internalization of 6E10 antibody. Cells were incubated with 6E10 antibody at 4 °C for 1 h. The temperature was then shifted to 37 °C, and cells were processed for immunofluorescence at the indicated times. *Scale bar* 10 µm. **c** Relative fluorescence intensity from 6E10 staining, showing the time course of APP degradation. **d** A zoom-in (the *square* in **b**) for the indicated times (0 and 20 min at 37 °C). Co-staining with anti-Rab4 antibody was used to visualize the APP within Rab4-positive endosomes involving in recycling. **e** Co-localization of 6E10 staining with Rab4 staining, as a guide to the APP level within recycling endosomes at the indicated times. **f** Cells transiently transfected with anti-FERMT2 in the presence or absence of siRab4. Extracts were analyzed by WB using anti-APP C-terminal, anti-FERMT2, anti-Rab4 or anti-actin antibodies

Since FERMT2 silencing reportedly enhances cell-surface CD39/CD73 levels by promoting their recycling to the plasma membrane [18], we also looked at whether FERMT2 has a similar effect on APP. To this end, we first explored the time course of APP degradation by using the alkalinizing drug bafilomycin A1 (BafA1) to block transport from late endosomes to lysosomes. We found that siFERMT2 significantly lowered the accumulation of mature APP upon BafA1 treatment (Fig. 5a)—suggesting that FERMT2 silencing may lead to a reduction in APP degradation by lysosomes.

We next assessed the impact of FERMT2 silencing on APP internalization. After incubation of HEK293-APP^695wt^ cells with 6E10 antibody at 4 °C, the time course of 6E10-APP complex endocytosis was monitored via immunofluorescence (Fig. 5b, c). After 20 min at 37 °C, APP was rapidly internalized into small vesicles at all levels of FERMT2 expression. Although internalization was unaffected, the remaining APP staining in vesicles was more intense in cells under-expressing FERMT2 than in control cells (respectively, 47 ± 16 versus 19 ± 9% of the initial APP staining observed at the cell surface before internalization). A similar observation was made at 40 min (32 ± 9 and 13 ± 7%, respectively). These data suggest that FERMT2 lowered lysosomal degradation of APP by favoring the recycling route and thus boosting cell surface levels of APP. Inhibition of β3-integrin reportedly promotes the activation of a RAB4-regulated pathway that (for example) diverts receptor tyrosine kinases (such as the VEGFR) from the degradative route back to the plasma membrane [4]. In order to establish whether a similar mechanism was operating for APP, we measured the effect of FERMT2 silencing on the APP recycling rate (i.e., the proportion of internalized APP colocalized with Rab4). We observed an increase in the proportion of Rab4+/APP+ endosomes; this indicated the induction of the APP recycling and was concordant with the above-mentioned accumulation of APP at the cell surface (Fig. 5d, e). Importantly, Rab4 expression did not change after siRNA-FERMT2 transfection. Lastly, we found that co-transfection of siRNA-FERMT2 with Rab4A-specific siRNA abolished the accumulation of mature APP (Fig. 5f). These data show that Rab4A is required for the induction of APP recycling after FERMT2 silencing. Taken as a whole, our results suggest that by facilitating APP recycling after endocytosis, FERMT2 controls the pool of mature APP available for cleavage by α-, β- and γ-secretases.

## Discussion

One can legitimately hypothesize that at least some of the genetic risk factors identified in GWASs have a role in APP metabolism and Aβ production. However, APP metabolism is a complex process and the underlying mechanisms have not been fully characterized yet. Literature data on these risk factors provide indirect clues but clearly cannot ascribe these genes with a genuine impact at a particular step in the pathophysiology of APP processing. Furthermore, a conventional gene-by-gene cell biology approach did not appear to be time- or cost-effective for investigating the 123 genes located within the GWAS-defined susceptibility loci. Hence, we developed an HCS assay for empirically testing multiple GWAS-identified genes and identifying modulators of APP metabolism.

Our genome-wide siRNA screening gave us an overview of the different factors likely to affect APP metabolism and thus enabled us to select the most significant APP modulators (i.e., the 5% throughout the genome showing the strongest variations). From among a total of 832 modulators, we identified 8 genes associated with the AD risk in the largest yet GWAS meta-analysis (the IGAP). Importantly, our approach is not fully exhaustive and it is impossible to exclude that genes involved in APP metabolism are localized outside of the peak defined by the GWAS but regulated by SNP associated with AD risk. Similarly, focusing on the 5% strongest variations may lead to exclude some genes of interest. For instance, although SORL1 was not included in the 832 best hits, under-expression of this gene had significant impact on mCherry and YFP signals when compared with non-targeting siRNA. Moreover, SNPs localized within *SORL1* showed an association with the CSF Aβ42 level (*p* = 0.007).

To validate the potential impact of these genes on APP metabolism in vivo, we assessed the association between SNPs in these genes and the CSF Aβ42 level in 2886 AD cases. Only FERMT2 was associated with significantly low levels of Aβ42 (*p* = 0.0006) after correction for multiple testing. The SNP rs62003531 most strongly associated with the Aβ42 level was in strong linkage disequilibrium (*r* ^2^ = 0.77) with the sentinel SNP rs17125944 (found to be associated with the AD risk in the IGAP’s GWAS meta-analysis). It is noteworthy that this sentinel polymorphism was also associated with Aβ42 levels (*p* = 0.03)—suggesting that both these SNPs represent the same genetic signal. Taken as a whole, these data indicate that FERMT2 regulates APP metabolism in general and Aβ loads in particular. Accordingly, we observed that FERMT2 silencing led to an increase in mature APP levels and Aβ secretion. Since we also observed that APP recycling was promoted by FERMT2 silencing through Rab4A-positive endosomes, our data strongly suggest that FERMT2 regulates APP recycling and its presence at the cell surface. The extracellular domain of APP is reportedly involved in cell–matrix adhesion and facilitates cell–cell adhesion via transcellular interactions [17]. Interaction between APP and integrin is required for adequate neurite outgrowth and contact guidance [24, 27]. Moreover, abnormal cleavage of APP might impair the protein’s functions in cell adhesion and migration [22]. Hence, the regulation of cell adhesion may be important in APP metabolism. Furthermore, FERMT2 expression is required for cell adhesion; recruitment of the focal adhesion kinase FAK and p130CAS is required for β3 integrin signaling [19, 25]. Several lines of evidence have established that integrin signaling controls the trafficking of other receptors and cargos [4]. For instance, inhibition of β3-integrin might promote the activation of a Rab4-regulated pathway that (for example) diverts receptor tyrosine kinases (such as the VEGFR) from their degradation route back to the plasma membrane [20]. These data suggest that FERMT2 has an integrin-dependent impact on mature APP levels via a similar mechanism. It is noteworthy that some of the main regulators of integrin signaling (e.g., β3-integrin, Src, paxillin and p130Cas) were also identified as modulators of APP metabolism in our HCS analysis (Supplemental Fig. 4).

Although FERMT2 expression appears to control cell surface levels of APP (which might modulate cell adhesion), our data also indicate that a FERMT2 under-expression may favor Aβ production. Interestingly, by using another independent, systematic approach, we have already suggested that low FERMT2 expression might contribute to the development of AD [8]. We reported that the rs7143400-T allele (associated with an increase in the AD risk and located within the FERMT2 3′ untranslated region [3′-UTR]) creates a perfect seed for miR-4504. Co-transfection of the rs7143400-T allele and miR-4504 resulted in lower luciferase activity (relative to the rs7143400-G allele co-transfected with the same miRNA). This observation indicated that a functional SNP within the FERMT2 3′-UTR region is associated with an increase in the AD risk and a potential miR-dependent decrease in FERMT2 expression. It is noteworthy that the rs7134400 SNP is in perfect linkage disequilibrium (*r* ^2^ = 1) with rs62003531, which exhibited the most significant association with the CSF level of Aβ42. Taken as a whole, our data suggest that the rs7134400 T allele is associated with an increased AD risk by lowering FERMT2 expression; in turn, this disrupts APP metabolism and favors Aβ production.

Lastly, we recently highlighted the fact that several GWAS-defined genes already known to be involved in the focal adhesion pathway (*Fak*, *Cass4* and *EPHA1*) are potential modulators of Tau toxicity in *Drosophila* [9]. Since genetic risk factors involved in cell adhesion signaling have been associated with Aβ production or Tau toxicity, we suggest that the characterization of these mechanisms should deepen our understanding of the link between amyloid and Tau in the AD process. FERMT2 might be located at the interface between these two hallmarks pathological processes because it has already been described as a modulator of Tau toxicity in *Drosophila* [23].

## Electronic supplementary material

Below is the link to the electronic supplementary material.

Supplementary material 1 (DOCX 5800 kb)
