# Supplementary material for: Genome-wide, high-content siRNA screening identifies the Alzheimer’s genetic risk factor FERMT2 as a major modulator of APP metabolism
Source: Acta Neuropathol. 2016 Dec 8;133(6):955–66. doi: 10.1007/s00401-016-1652-z (PMC5427165; doi:10.1007/s00401-016-1652-z)
Supplement: Supplementary file 1 — Supplementary material 1 (DOCX 5800 kb) [file 401_2016_1652_MOESM1_ESM.docx]

**Supplemental Fig 1**

**a,** and **b,** representative Western blot showing the impact of 6 hours treatment on HEK293-APP^695WT^ (proteasome inhibitor and γ-secretase inhibitor respectively). Graph show the impact of these treatments on mCherry and YFP fluorescence intensity using HEK293-mCherry-APP^695wt^-YFP cell line. The effects obtained in both cellular models were consistent with a specific increase of YFP fluorescent intensity (corresponding to APP-CTF) after the inhibition of the γ-secretase activity. **c**, Formulation used for the SSMD measurement of the strength of the difference between two controls (PSEN1 and APP). **d**, Graph shows β scores for 9 test plates based on the analysis of 200 or 1,000 cells per well. **e**, Location of siRNA controls and genome-wide siRNA bank on a 384-well plate. The layouts attempt to systematically decrease the edge bias by alternating the spatial position of the controls so that they appear in equal quantity on each of the rows and available columns. **f**, Graph shows β scores for each plate. Tree plates (in red) showed β score below 3 and have been removed from the analysis.


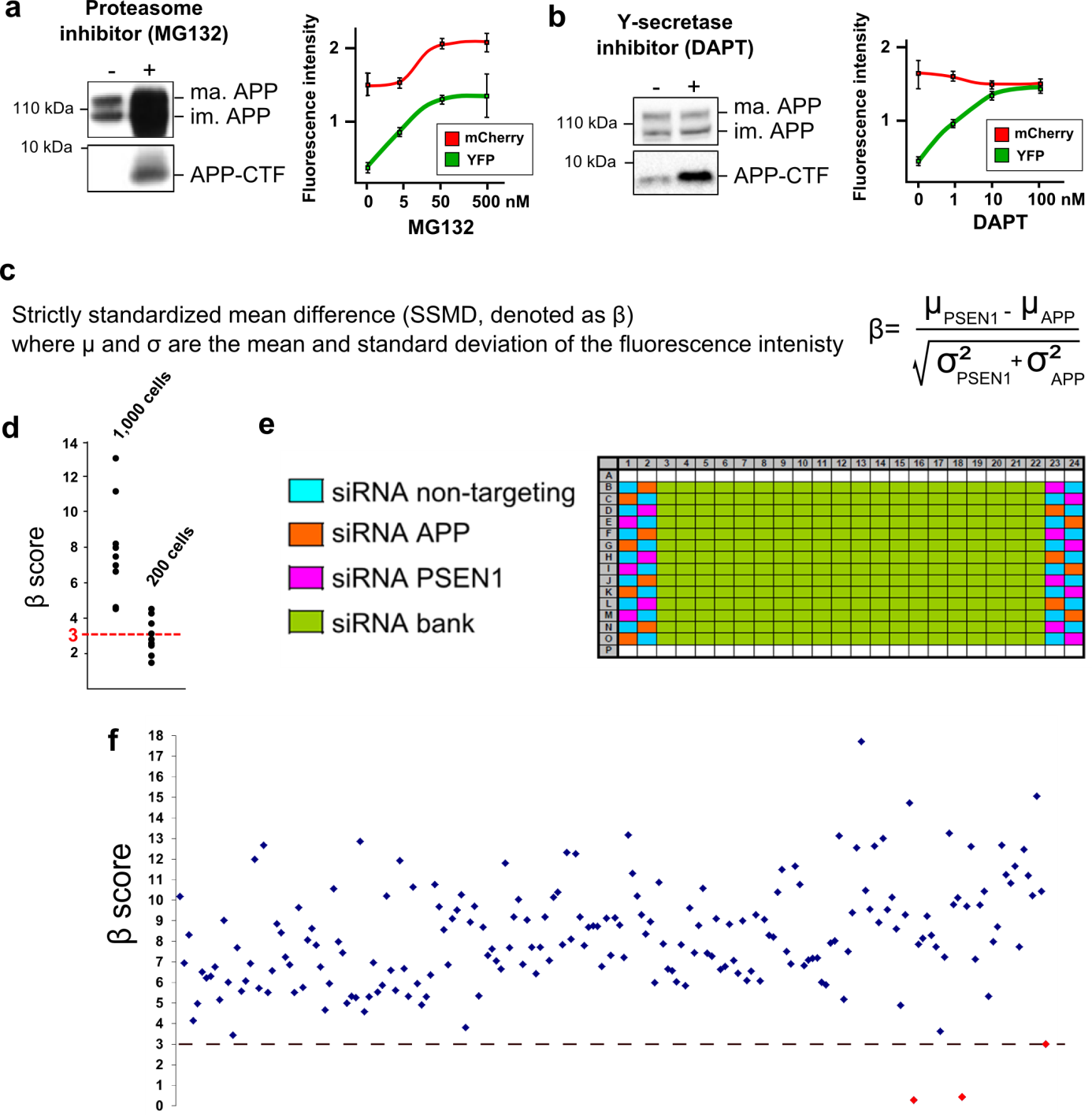


**Supplemental Fig 2**

Quantification showing the mCherry and YFP intensity variation (fold change) based on HCS data for ADAM10, PSEN1, BACE1 and SORL1 siRNA compared with Non-Targeting siRNA. Histograms indicate the means ± S.D. * p<0.05, non-parametric test.


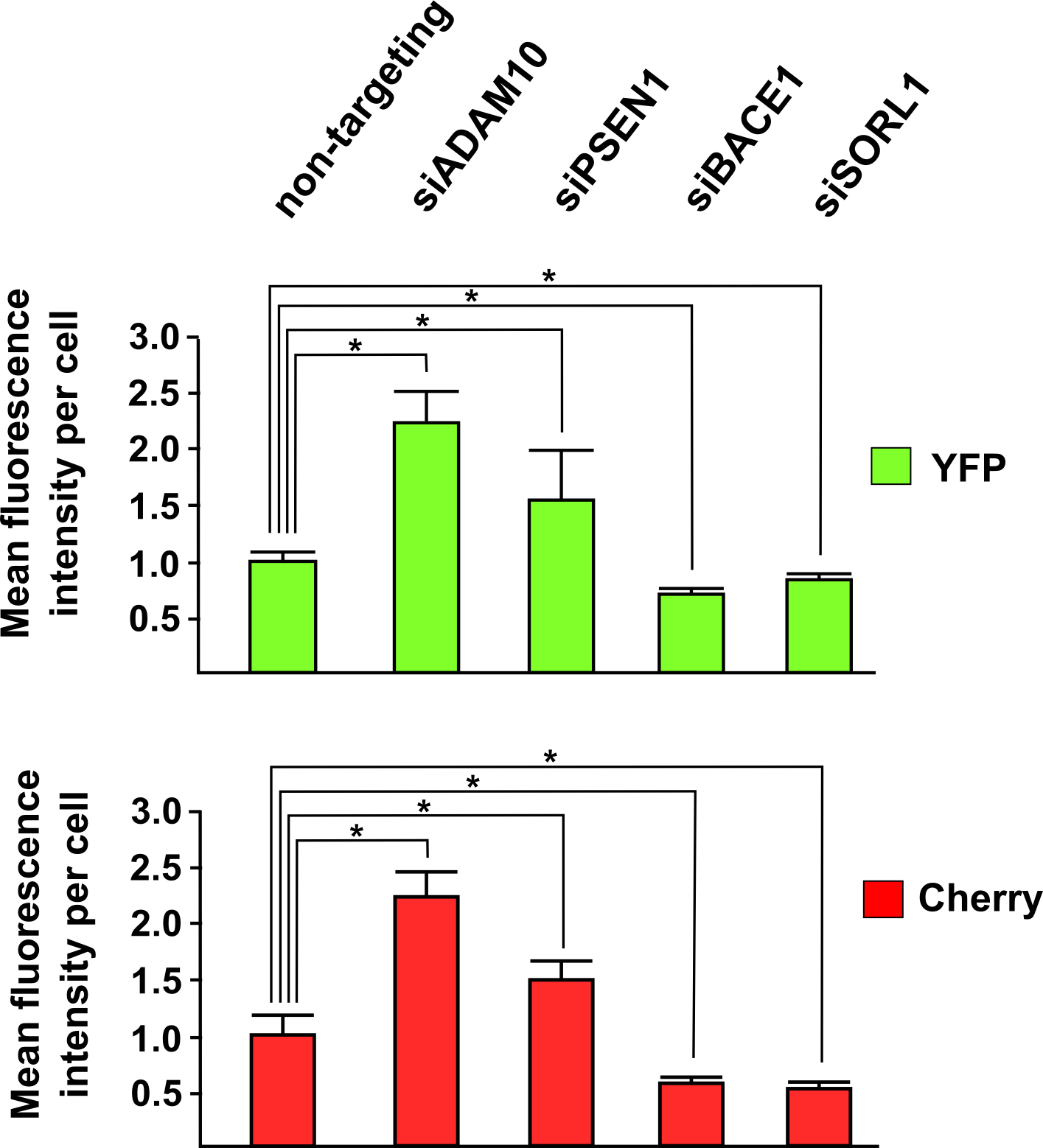


**Supplemental Fig 3**

Protein-protein interaction (direct interaction only) network between the 832 hits identified as modulators of the APP metabolism. This network was obtained using the Ingenuity Pathway Analysis (IPA) software. Of note, APP (Red) appears to be the protein showing the highest number of interaction with other proteins within this network


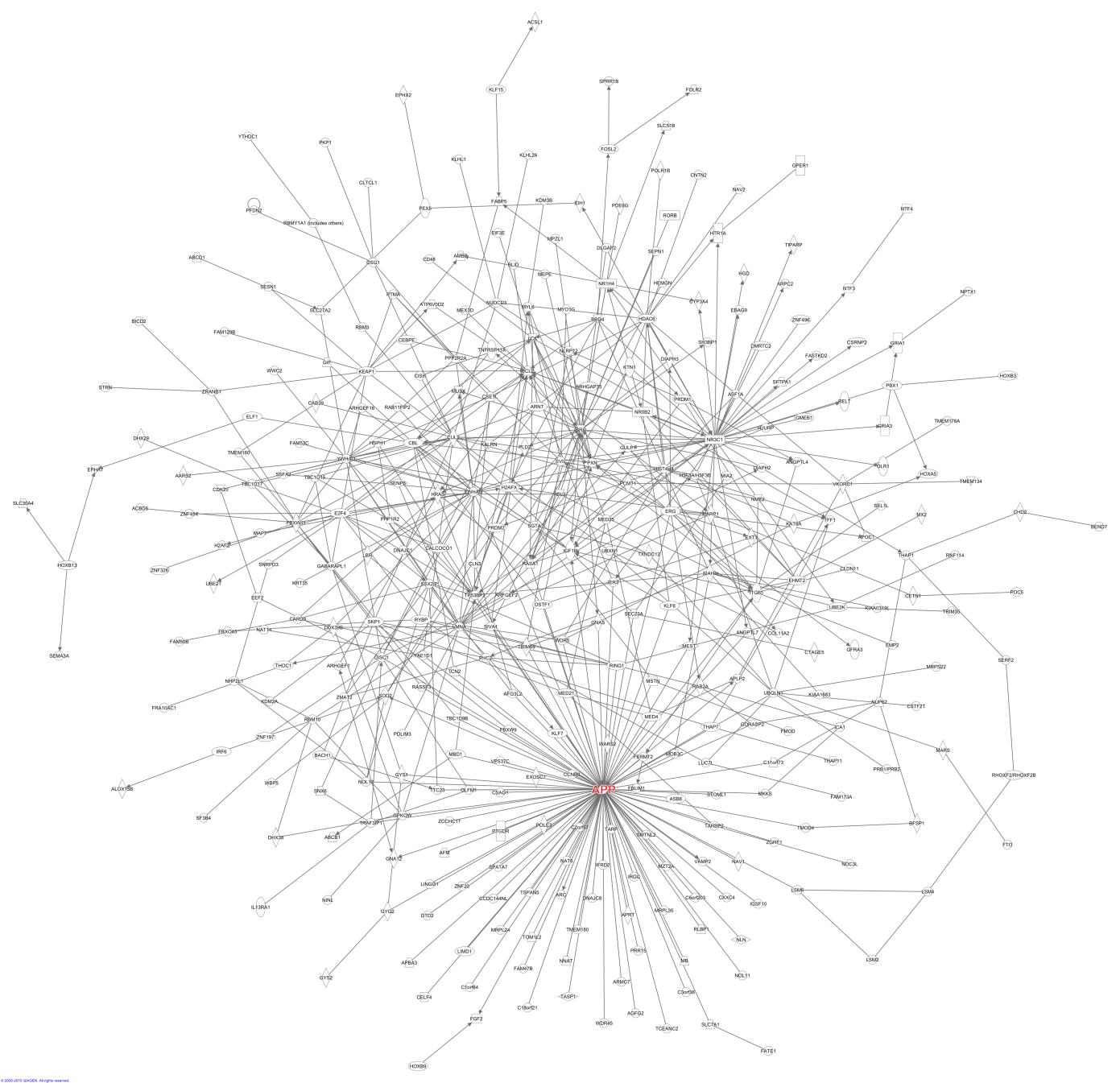


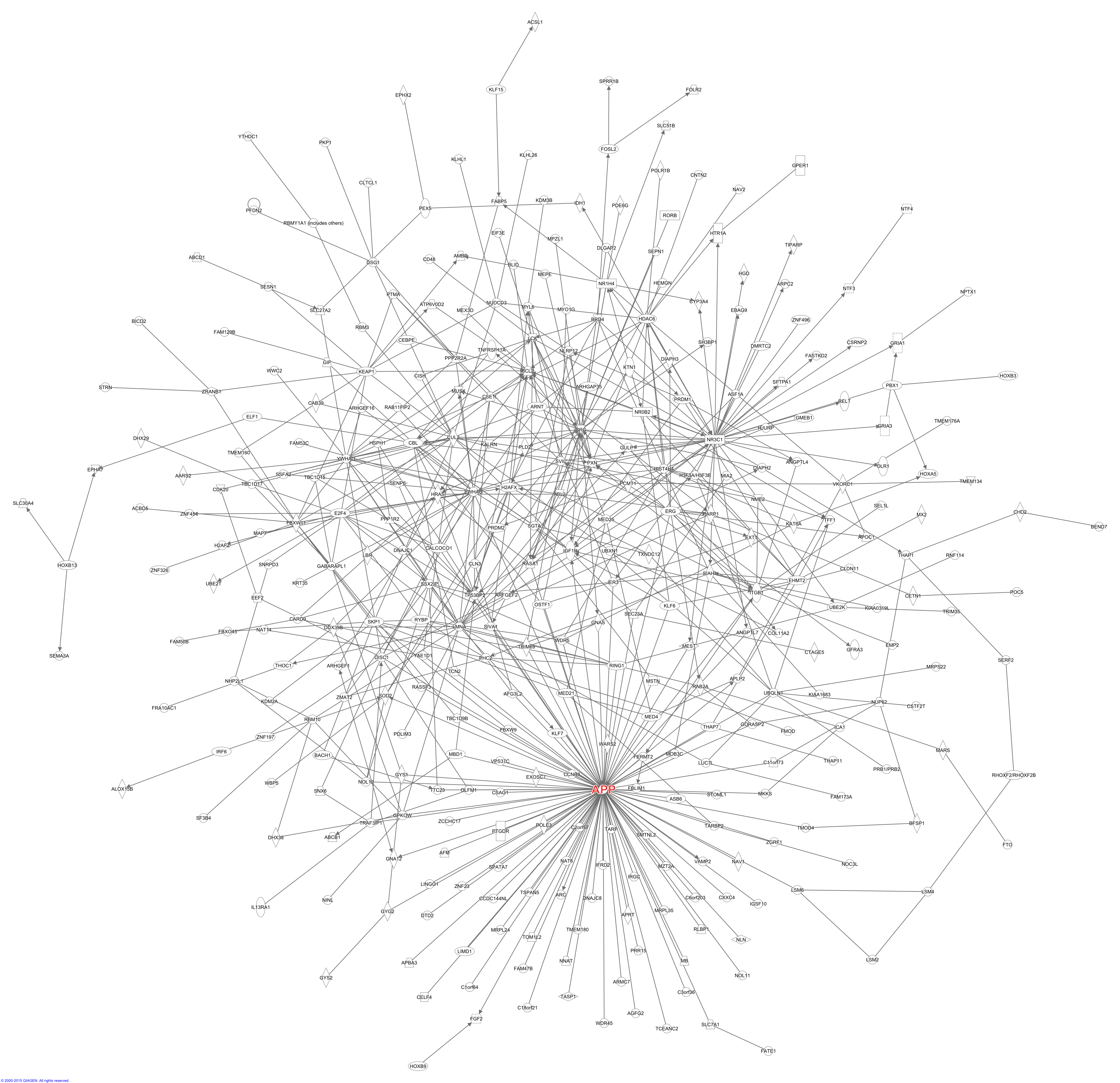


**Supplemental Fig 4**

In green are the hits identified by HCS among the focal adhesion pathways according to KEGG pathway database (RTK, Receptor Tyrosine Kinase).


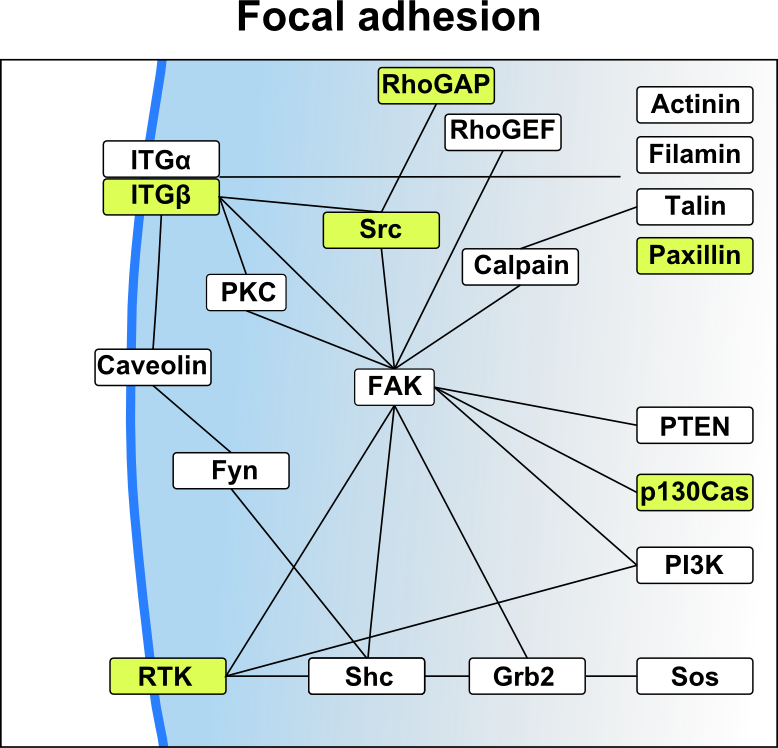


**Supplemental Fig 5**

Quantification showing the mCherry and YFP intensity variation (fold change) after transfection of four different siRNA targeting FERMT2 independently. Three of them (#1, #2, #4) were associated with a significant increase of both mCherry and YFP signals consistently with the effect of the SMART Poll used for HCS. Histograms indicate the means ± S.D. * p<0.05, non-parametric test.


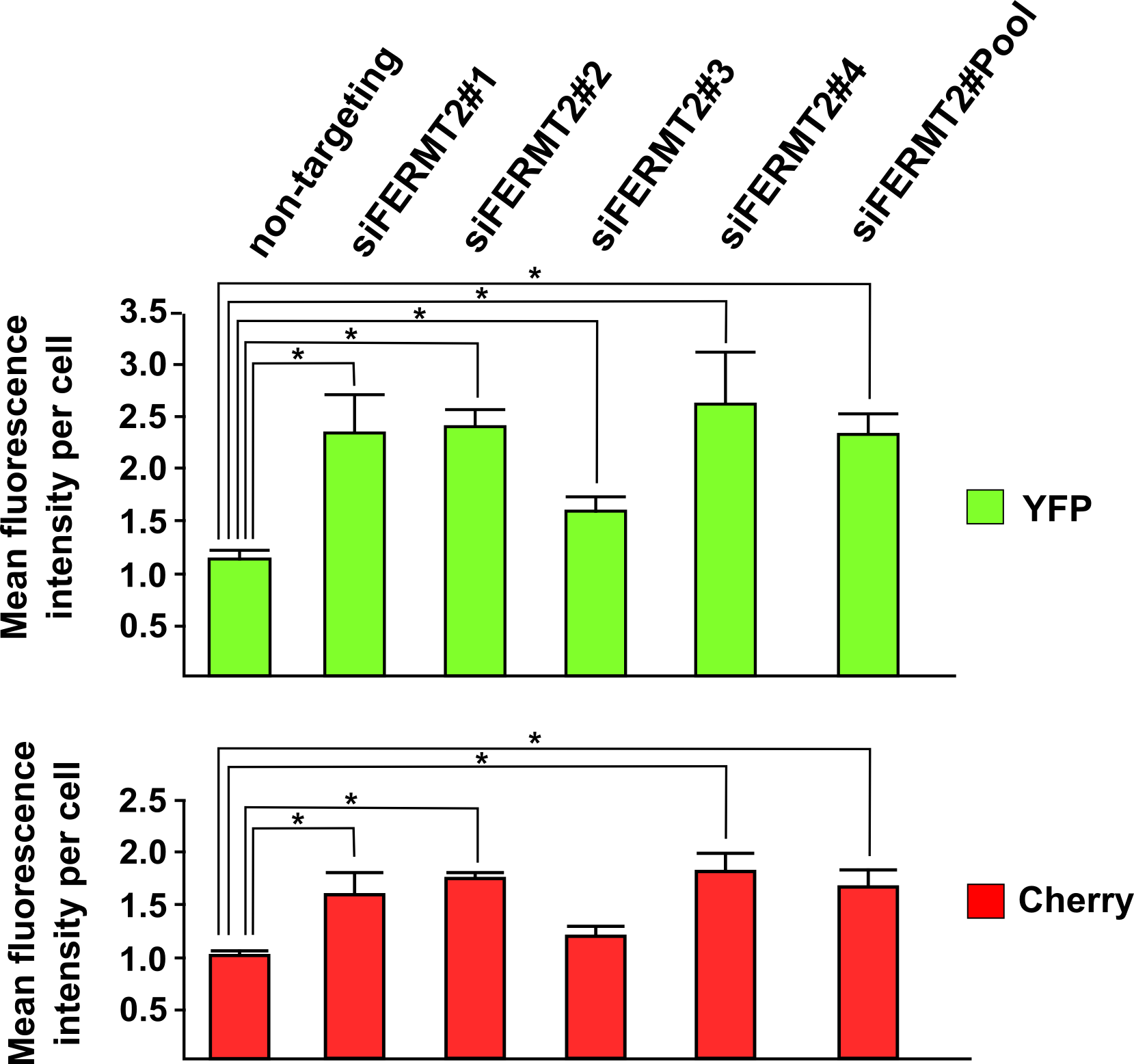


**Supplemental Fig 6**

**a**, Impact of FERMT2 silencing on APP metabolism in 293HEK-APP^695WT^ cell line. Cells transiently transfected with four different anti-FERMT2 or non-targeting siRNA were analyzed by WB using anti-APP Cterminal, anti-FERMT2 and anti-Actin antibodies. ma. APP, mature APP; im. APP, immature APP. **b**, Shown are densitometric analysis and quantification of WB in three independent experiments. **c**, Conditioned medium was analyzed by AlphaLISA to detect secreted Aβ1-X. Histograms indicate the means ± S.D. a.u., arbitrary units. * p<0.05, non-parametric test. Of note, #1, #2 and #4 siRNA showed a consistent effect on mature APP and Aβ level with the SMART Pool siRNA.


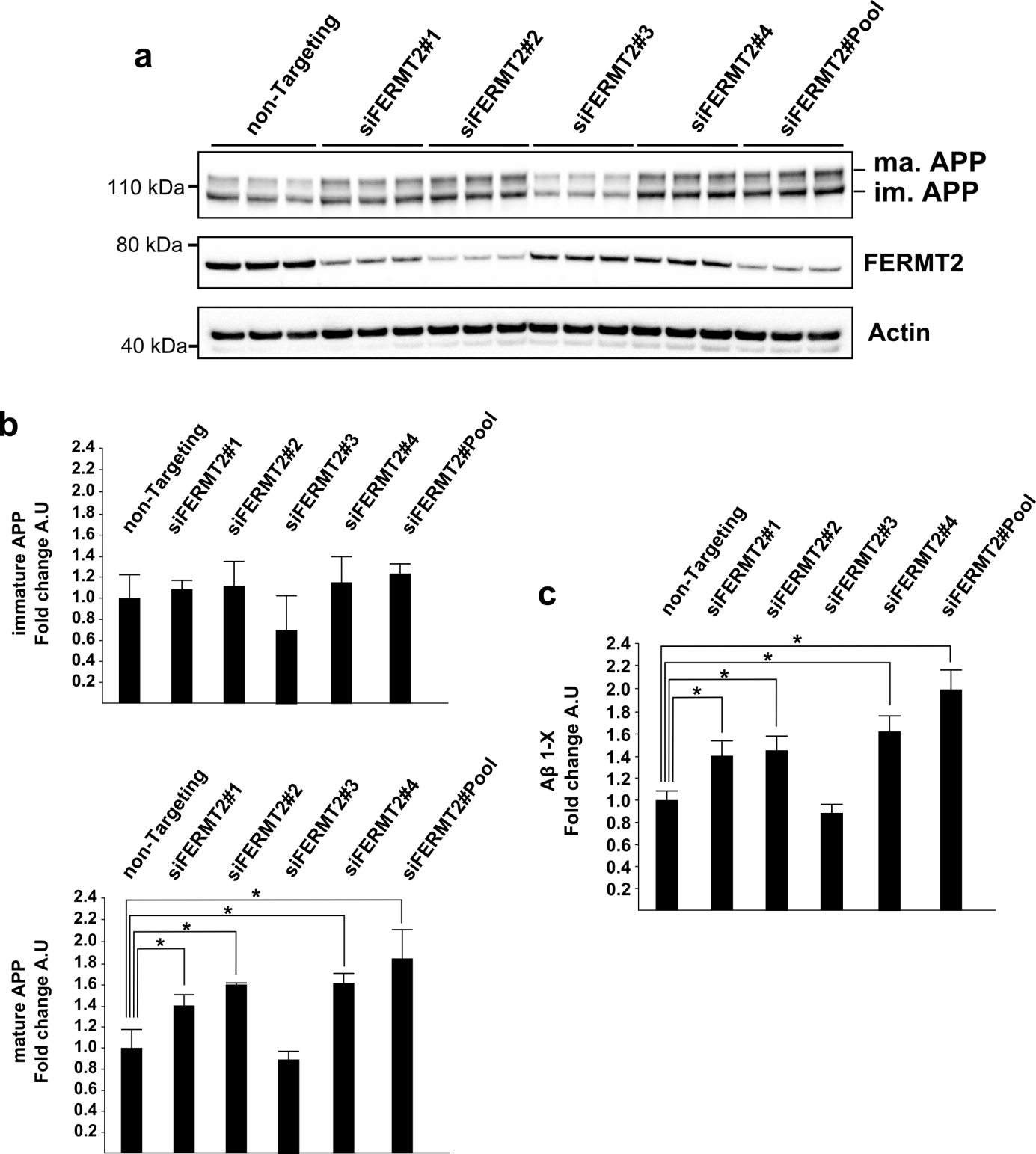


**Supplemental Fig 7**

Cell-surface-biotinylated proteins from HEK293 cells transfected with siFERMT2 or non-targeting siRNA. Cells extracts were precipitated with immobilized avidin and analyzed by WB using antibodies directed against APP, FERMT2, Actin (intra-cellular marker), and Na-K-ATPase α1 (cell surface marker). ma. APP, mature APP; im. APP, immature APP. Shown are representative WB in three independent experiments in HEK293 cells line expressing cherry-APP-YFP (**a**) or endogenous APP (**b**).


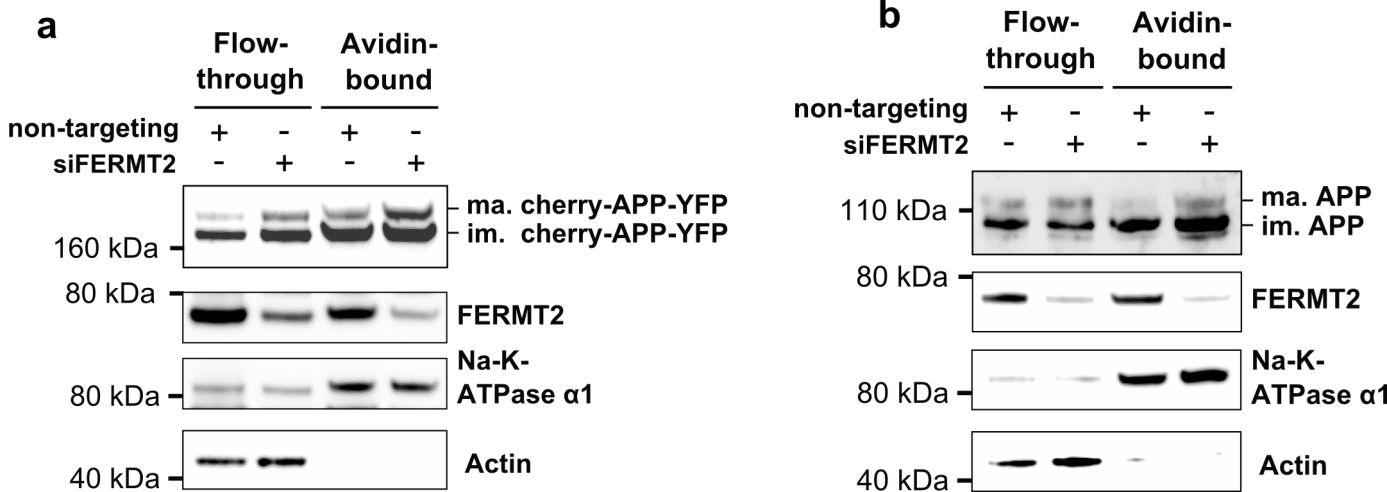


**Table 1**

Variation of mCherry and YFP signals (Log2 of fold change) for the best 5% hits (n=832) exhibiting the strongest mCherry variations. s.d. standard deviation.

| Gene | YFP | |  | mCherry | |
| --- | --- | --- | --- | --- | --- |
|  | Log2 Fold change | sd |  | Log2 Fold change | sd |
| MGC35295 | **2.3553** | 0.2642 |  | **2.6989** | 0.2010 |
| TACR1 | **2.7822** | 0.3813 |  | **2.6824** | 0.3549 |
| SEMA3F | **2.0216** | 0.1466 |  | **2.5065** | 0.1632 |
| CTXN1 | **2.0631** | 0.1649 |  | **2.5034** | 0.0735 |
| YWHAG | **4.1691** | 0.3402 |  | **2.4894** | 0.2019 |
| FLJ20522 | **1.7997** | 0.1108 |  | **2.4407** | 0.0766 |
| SPATA7 | **2.1599** | 0.1644 |  | **2.4072** | 0.1672 |
| NNAT | **2.1700** | 0.1926 |  | **2.4041** | 0.1512 |
| CNTN2 | **2.5326** | 0.1013 |  | **2.3935** | 0.0123 |
| DKFZP434G072 | **2.1710** | 0.1715 |  | **2.3751** | 0.1054 |
| TGIF2LX | **2.1801** | 0.0536 |  | **2.3659** | 0.1033 |
| NTF3 | **2.0999** | 0.1225 |  | **2.3563** | 0.1081 |
| RAB11-FIP2 | **1.7048** | 0.0929 |  | **2.3462** | 0.1584 |
| PRB1 | **1.6907** | 0.2692 |  | **2.3354** | 0.2047 |
| NUP62 | **1.9301** | 0.1024 |  | **2.3149** | 0.1020 |
| PDLIM3 | **2.0022** | 0.1516 |  | **2.2813** | 0.2043 |
| USP43 | **1.9854** | 0.2421 |  | **2.2693** | 0.1409 |
| TNFRSF19L | **1.5363** | 0.0662 |  | **2.2603** | 0.0245 |
| DEFA3 | **1.9933** | 0.1049 |  | **2.2405** | 0.2035 |
| C13ORF22 | **2.1641** | 0.1025 |  | **2.2246** | 0.1449 |
| FLJ35779 | **1.3221** | 0.1873 |  | **2.2227** | 0.2210 |
| LST1 | **2.2238** | 0.4108 |  | **2.1962** | 0.3674 |
| GPKOW | **2.5025** | 1.6503 |  | **2.1906** | 1.0784 |
| ZFY | **1.6032** | 0.2382 |  | **2.1760** | 0.1921 |
| POLR1B | **1.8244** | 0.4103 |  | **2.1692** | 0.4409 |
| KRTAP13-2 | **1.8643** | 0.1010 |  | **2.1581** | 0.0556 |
| DHX29 | **1.6308** | 0.4197 |  | **2.1493** | 0.3668 |
| FLJ14627 | **1.6245** | 0.0547 |  | **2.1342** | 0.1339 |
| CMIP | **1.7299** | 0.1007 |  | **2.1310** | 0.0875 |
| H2AFX | **1.9276** | 0.3086 |  | **2.1292** | 0.2743 |
| UNQ9217 | **1.5877** | 0.1418 |  | **2.1272** | 0.1190 |
| GAS | **1.7915** | 0.1416 |  | **2.1231** | 0.2285 |
| NTF5 | **1.4098** | 0.1706 |  | **2.1203** | 0.0910 |
| XAB1 | **1.8631** | 0.1853 |  | **2.1045** | 0.1073 |
| DMRTC2 | **2.3014** | 0.0607 |  | **2.0973** | 0.0915 |
| FEV | **1.7570** | 0.2518 |  | **2.0945** | 0.2744 |
| SRCRB4D | **1.3595** | 0.1054 |  | **2.0835** | 0.1708 |
| SOX12 | **2.5590** | 0.2757 |  | **2.0768** | 0.0221 |
| LOC340069 | **1.8398** | 0.1194 |  | **2.0753** | 0.1162 |
| FLJ22160 | **1.1749** | 0.0927 |  | **2.0735** | 0.1169 |
| TIPARP | **1.6606** | 0.2724 |  | **2.0645** | 0.2445 |
| LSM4 | **1.7545** | 0.1966 |  | **2.0598** | 0.2538 |
| SN | **1.7880** | 0.2314 |  | **2.0483** | 0.2383 |
| ALOX15B | **1.3413** | 0.1486 |  | **2.0465** | 0.1376 |
| FLJ25811 | **1.3905** | 0.2012 |  | **2.0437** | 0.1598 |
| CRLF1 | **1.3908** | 0.2027 |  | **2.0406** | 0.2514 |
| FLJ46072 | **1.3252** | 0.0585 |  | **2.0395** | 0.0495 |
| DOC2A | **1.1985** | 0.0376 |  | **2.0324** | 0.0938 |
| LOC401546 | **1.2544** | 0.1660 |  | **2.0298** | 0.0934 |
| TIAF1 | **1.5809** | 0.1899 |  | **2.0290** | 0.2125 |
| C1orf164 | **1.7842** | 0.4501 |  | **2.0275** | 0.5202 |
| PRAMEF8 | **1.6539** | 0.0951 |  | **2.0246** | 0.1769 |
| CKLFSF8 | **1.8834** | 0.0615 |  | **2.0219** | 0.0693 |
| ELF1 | **1.7442** | 0.3228 |  | **2.0215** | 0.1917 |
| FLJ13621 | **1.4243** | 0.2341 |  | **2.0199** | 0.2214 |
| FLJ10490 | **1.5954** | 0.1063 |  | **2.0140** | 0.1029 |
| SURB7 | **1.4287** | 0.2163 |  | **2.0085** | 0.1424 |
| OR3A4 | **1.8931** | 0.1750 |  | **2.0058** | 0.2712 |
| COL11A2 | **1.5445** | 0.1797 |  | **2.0054** | 0.1407 |
| C9ORF88 | **1.0929** | 0.1296 |  | **2.0036** | 0.0800 |
| FLJ25076 | **1.7356** | 0.1233 |  | **1.9973** | 0.1612 |
| RBM12 | **1.3243** | 0.0706 |  | **1.9919** | 0.1079 |
| COP | **2.0375** | 0.1605 |  | **1.9869** | 0.0736 |
| MGC10233 | **1.3433** | 0.1055 |  | **1.9863** | 0.0599 |
| PDHA2 | **1.3373** | 0.2750 |  | **1.9851** | 0.2830 |
| C18ORF4 | **1.6967** | 0.1560 |  | **1.9836** | 0.0791 |
| BRD4 | **1.9014** | 0.2300 |  | **1.9766** | 0.1579 |
| SNAPC4 | **1.7423** | 0.1051 |  | **1.9746** | 0.1227 |
| OR6C3 | **1.7936** | 0.2278 |  | **1.9661** | 0.1694 |
| HIP2 | **1.4943** | 0.1259 |  | **1.9627** | 0.0999 |
| RGN | **1.7501** | 0.0782 |  | **1.9595** | 0.0982 |
| SYTL4 | **1.6274** | 0.1884 |  | **1.9516** | 0.1316 |
| AKR7A3 | **2.0289** | 0.2179 |  | **1.9491** | 0.1863 |
| HDAC6 | **1.5909** |  |  | **1.9367** |  |
| ARC | **1.1703** | 0.3266 |  | **1.9362** | 0.2548 |
| GNA11 | **1.5068** | 0.0556 |  | **1.9241** | 0.1374 |
| OR8B8 | **1.5618** | 0.1206 |  | **1.9217** | 0.1881 |
| KIAA0117 | **1.4611** | 0.1893 |  | **1.9171** | 0.0840 |
| HLA-G | **1.8550** | 0.0753 |  | **1.9146** | 0.2288 |
| TFF1 | **1.6279** | 0.0462 |  | **1.9129** | 0.0581 |
| TRIM35 | **1.8359** | 0.2982 |  | **1.9056** | 0.5009 |
| IL17C | **1.2791** |  |  | **1.9014** |  |
| HLA-DRB3 | **1.5909** | 0.1092 |  | **1.8984** | 0.1768 |
| BPNT1 | **1.3836** | 0.0673 |  | **1.8954** | 0.1847 |
| YT521 | **1.1648** | 0.0386 |  | **1.8954** | 0.0380 |
| STARD4 | **1.6833** | 0.0603 |  | **1.8934** | 0.0759 |
| MO25 | **1.7608** | 0.1259 |  | **1.8906** | 0.2099 |
| RAP140 | **1.3728** | 0.0354 |  | **1.8843** | 0.0602 |
| LOC643923 | **1.6286** | 0.1526 |  | **1.8842** | 0.2658 |
| DKFZP762K222 | **1.4781** | 0.1446 |  | **1.8753** | 0.1652 |
| FLJ32871 | **1.2336** | 0.0679 |  | **1.8733** | 0.0459 |
| KIF21A | **1.2758** | 0.4446 |  | **1.8732** | 0.6415 |
| ZNF213 | **1.3027** | 0.1376 |  | **1.8656** | 0.1990 |
| SLC29A3 | **1.5593** | 0.1284 |  | **1.8638** | 0.0275 |
| STOML1 | **1.6427** | 0.0634 |  | **1.8621** | 0.0605 |
| LCK | **1.1699** | 0.1028 |  | **1.8612** | 0.2183 |
| MGC35154 | **1.3949** | 0.3510 |  | **1.8570** | 0.3172 |
| FLJ31121 | **1.4593** | 0.2079 |  | **1.8531** | 0.1500 |
| SF3B4 | **1.8794** | 0.1777 |  | **1.8490** | 0.1472 |
| LOC401250 | **1.1939** | 0.0669 |  | **1.8483** | 0.1838 |
| WDRX1 | **1.1976** | 0.1468 |  | **1.8447** | 0.2277 |
| PEPP-2 | **1.1865** | 0.1106 |  | **1.8409** | 0.2519 |
| KRTHA5 | **1.3029** | 0.3133 |  | **1.8394** | 0.3131 |
| MGC27121 | **1.9354** | 0.2285 |  | **1.8365** | 0.1342 |
| GPR30 | **1.5928** | 0.1025 |  | **1.8321** | 0.4029 |
| GJA4 | **1.2603** | 0.1827 |  | **1.8314** | 0.1851 |
| IL17E | **1.1570** | 0.0260 |  | **1.8267** | 0.1644 |
| MGC2463 | **1.2909** | 0.0382 |  | **1.8259** | 0.0173 |
| FGF2 | **2.1508** | 0.0500 |  | **1.8223** | 0.0270 |
| BRUNOL4 | **1.4295** | 0.1777 |  | **1.8203** | 0.0507 |
| C2 | **1.5366** | 0.0334 |  | **1.8157** | 0.0651 |
| FLJ32384 | **1.1047** | 0.2934 |  | **1.8145** | 0.4070 |
| ZNF496 | **1.3080** | 0.1618 |  | **1.8128** | 0.1598 |
| GPR68 | **1.2830** | 0.0846 |  | **1.8080** | 0.1035 |
| ARPC2 | **1.4876** | 0.1003 |  | **1.8070** | 0.1349 |
| AKAP8 | **1.4083** | 0.1522 |  | **1.8027** | 0.2002 |
| BRI3BP | **1.0524** | 0.0968 |  | **1.8026** | 0.0923 |
| KTN1 | **1.7857** | 0.1244 |  | **1.7949** | 0.2036 |
| ZNF454 | **1.2675** | 0.4458 |  | **1.7929** | 0.6355 |
| TOR3A | **1.3031** | 0.1821 |  | **1.7918** | 0.1737 |
| KEAP1 | **1.2866** | 0.1199 |  | **1.7896** | 0.1307 |
| C5ORF19 | **1.7024** | 0.1237 |  | **1.7854** | 0.1215 |
| TRAM1L1 | **1.0879** | 0.1818 |  | **1.7837** | 0.2500 |
| MMAA | **1.1928** | 0.2099 |  | **1.7800** | 0.1025 |
| C9ORF74 | **0.9570** | 0.0831 |  | **1.7772** | 0.0990 |
| MAP3K9 | **1.0458** | 0.1521 |  | **1.7643** | 0.4136 |
| RORB | **1.8499** | 0.0121 |  | **1.7617** | 0.1077 |
| FLJ31846 | **1.2828** | 0.2236 |  | **1.7606** | 0.1501 |
| C7ORF36 | **1.4463** | 0.0449 |  | **1.7602** | 0.0893 |
| LUC7L | **1.4560** | 0.1840 |  | **1.7580** | 0.3551 |
| C10ORF117 | **1.1828** | 0.3279 |  | **1.7573** | 0.2612 |
| RBM6 | **1.2200** | 0.1773 |  | **1.7568** | 0.1166 |
| NEK7 | **1.6874** | 0.1152 |  | **1.7563** | 0.1444 |
| FLJ20847 | **1.0363** | 0.2560 |  | **1.7560** | 0.2139 |
| SLC25A14 | **1.8109** | 0.1428 |  | **1.7533** | 0.0602 |
| FOXE1 | **1.6262** | 0.1725 |  | **1.7461** | 0.2251 |
| EXOSC7 | **1.3686** | 0.1481 |  | **1.7428** | 0.1236 |
| ABL2 | **2.0259** | 0.0147 |  | **1.7415** | 0.0867 |
| FLJ25416 | **1.4704** | 0.1166 |  | **1.7410** | 0.1312 |
| CSTF2T | **1.0791** | 0.2105 |  | **1.7380** | 0.2456 |
| AARSL | **1.0694** | 0.0930 |  | **1.7380** | 0.0926 |
| DKFZP586L0724 | **1.2220** | 0.1005 |  | **1.7373** | 0.0646 |
| KRTAP19-2 | **1.6159** | 0.9069 |  | **1.7286** | 0.8944 |
| REP15 | **0.9863** | 0.0933 |  | **1.7259** | 0.1131 |
| FGF22 | **1.4466** | 0.2777 |  | **1.7241** | 0.2946 |
| LOC112703 | **1.6520** | 0.1380 |  | **1.7218** | 0.2418 |
| PRDM4 | **1.1918** | 0.2362 |  | **1.7213** | 0.3893 |
| CCDC7 | **1.5418** | 0.4793 |  | **1.7209** | 0.3360 |
| LSM2 | **1.3736** | 0.1176 |  | **1.7186** | 0.2016 |
| CD6 | **1.3411** | 0.0553 |  | **1.7177** | 0.0637 |
| FLJ20512 | **1.3034** | 0.1892 |  | **1.7156** | 0.3397 |
| TACC2 | **1.0029** | 0.0436 |  | **1.7141** | 0.1084 |
| CCL28 | **1.9237** | 0.0496 |  | **1.7139** | 0.1130 |
| SP6 | **1.3348** | 0.1030 |  | **1.7136** | 0.1193 |
| HTR1A | **1.7727** | 0.1920 |  | **1.7108** | 0.2710 |
| POLR3B | **1.4284** | 0.2315 |  | **1.7103** | 0.2759 |
| CXXC4 | **1.0258** | 0.1243 |  | **1.7064** | 0.2799 |
| SYT3 | **1.1728** | 0.2281 |  | **1.7050** | 0.2616 |
| MGEA6 | **1.1050** | 0.0752 |  | **1.7007** | 0.0837 |
| PCP4 | **1.5985** | 0.2334 |  | **1.6997** | 0.2869 |
| KALRN | **1.2425** | 0.1307 |  | **1.6954** | 0.1892 |
| HSPC129 | **1.2289** | 0.1451 |  | **1.6934** | 0.0505 |
| MGC14560 | **1.2664** | 0.1022 |  | **1.6932** | 0.0856 |
| MAP4K5 | **1.3686** | 0.0577 |  | **1.6923** | 0.4003 |
| RAB3IP | **0.9466** | 0.1555 |  | **1.6922** | 0.3401 |
| KRTHA2 | **1.4153** | 0.0796 |  | **1.6915** | 0.2260 |
| NHP2L1 | **2.1343** | 0.0505 |  | **1.6906** | 0.0963 |
| D6S2654E | **1.1650** | 0.1216 |  | **1.6902** | 0.0835 |
| ACSL1 | **1.6732** | 0.1865 |  | **1.6888** | 0.1535 |
| EPHA7 | **0.7131** | 0.1142 |  | **1.6868** | 0.1804 |
| KIAA1627 | **1.2370** | 0.1224 |  | **1.6863** | 0.0827 |
| C9ORF20 | **1.2113** | 0.0853 |  | **1.6845** | 0.0741 |
| BRCC2 | **1.3627** | 0.3732 |  | **1.6829** | 0.4315 |
| HSPC138 | **1.0767** | 0.2399 |  | **1.6817** | 0.2460 |
| LOC728656 | **1.2793** | 0.0748 |  | **1.6807** | 0.1459 |
| IGSF10 | **1.3352** | 0.1779 |  | **1.6796** | 0.0611 |
| ZNF23 | **1.4663** | 0.3117 |  | **1.6788** | 0.3803 |
| FLJ32332 | **1.6391** |  |  | **1.6760** |  |
| CMKOR1 | **1.3051** | 0.3470 |  | **1.6756** | 0.2475 |
| FLJ35782 | **1.0649** | 0.2370 |  | **1.6751** | 0.2608 |
| FLJ39237 | **1.2069** | 0.5696 |  | **1.6748** | 0.5529 |
| PLEKHC1 | **1.1934** | 0.3417 |  | **1.6722** | 0.2855 |
| NR1H4 | **1.9133** | 0.1857 |  | **1.6716** | 0.0437 |
| SERF2 | **1.4205** | 0.2644 |  | **1.6700** | 0.2122 |
| OFCC1 | **1.4327** | 0.1254 |  | **1.6694** | 0.0893 |
| PIR | **1.6442** | 0.1610 |  | **1.6692** | 0.1766 |
| FLJ42461 | **1.0527** | 0.1959 |  | **1.6686** | 0.1307 |
| MGC33947 | **1.1248** | 0.2115 |  | **1.6631** | 0.2812 |
| RBM3 | **1.3594** | 0.0486 |  | **1.6628** | 0.2177 |
| FLJ12572 | **1.3036** | 0.1908 |  | **1.6628** | 0.1299 |
| CDT6 | **1.6421** | 0.2314 |  | **1.6595** | 0.2315 |
| MGC52057 | **1.1209** | 0.0871 |  | **1.6575** | 0.0760 |
| ZBTB39 | **1.5495** | 0.1072 |  | **1.6562** | 0.1808 |
| BOMB | **1.1044** | 0.0584 |  | **1.6560** | 0.0440 |
| JMJD1B | **0.7542** | 0.0526 |  | **1.6524** | 0.1287 |
| DKFZP762E1312 | **1.5168** | 0.0536 |  | **1.6495** | 0.0796 |
| GUCA2B | **1.3708** | 0.0302 |  | **1.6491** | 0.0442 |
| MTCH1 | **1.8902** | 0.4227 |  | **1.6482** | 0.1598 |
| FKBP3 | **1.2367** | 0.2526 |  | **1.6444** | 0.2470 |
| FRBZ1 | **0.9912** | 0.1510 |  | **1.6442** | 0.2262 |
| TXNDC12 | **1.0867** | 0.1539 |  | **1.6439** | 0.1553 |
| SEMA4B | **1.0733** | 0.5895 |  | **1.6438** | 0.7153 |
| MOBKL2C | **1.0935** | 0.0601 |  | **1.6432** | 0.0955 |
| FLJ21749 | **0.9830** | 0.0402 |  | **1.6430** | 0.0942 |
| DISC1 | **1.0090** | 0.0573 |  | **1.6429** | 0.2124 |
| DKFZP564B167 | **1.0163** | 0.1558 |  | **1.6377** | 0.1700 |
| OR10S1 | **2.1571** | 0.2947 |  | **1.6373** | 0.0941 |
| MGC11332 | **1.1586** | 0.0654 |  | **1.6357** | 0.1179 |
| AMBP | **1.4381** | 0.0513 |  | **1.6354** | 0.0615 |
| MGC33094 | **1.0762** | 0.1314 |  | **1.6334** | 0.2439 |
| LBR | **1.3982** | 0.2668 |  | **1.6332** | 0.2727 |
| MGC3036 | **1.0445** | 0.0913 |  | **1.6312** | 0.0715 |
| NDUFAF1 | **1.4155** | 0.2125 |  | **1.6306** | 0.2661 |
| PFDN2 | **1.0643** | 0.2250 |  | **1.6294** | 0.1789 |
| K6IRS2 | **1.2958** | 0.2457 |  | **1.6277** | 0.2791 |
| TMEM14C | **1.2043** | 0.2981 |  | **1.6267** | 0.2290 |
| C6ORF203 | **1.2002** | 0.5250 |  | **1.6250** | 0.3393 |
| ZNF432 | **1.3944** | 0.1709 |  | **1.6233** | 0.1472 |
| FLJ35725 | **1.4145** | 0.1348 |  | **1.6232** | 0.0636 |
| MX2 | **1.6418** | 0.1114 |  | **1.6232** | 0.1297 |
| IGF1R | **1.4267** | 0.1802 |  | **1.6209** | 0.1909 |
| HOXB9 | **1.5276** | 0.1272 |  | **1.6194** | 0.0857 |
| TMEPAI | **1.3276** | 0.8640 |  | **1.6175** | 0.9100 |
| VTCN1 | **1.0408** | 0.0849 |  | **1.6159** | 0.0733 |
| CSE1L | **1.3296** | 0.1207 |  | **1.6152** | 0.1148 |
| MLP | **1.0911** | 0.0640 |  | **1.6143** | 0.0478 |
| ARPP-21 | **1.4662** | 0.2289 |  | **1.6122** | 0.0898 |
| OTOS | **1.6185** | 0.1440 |  | **1.6088** | 0.1513 |
| GPR20 | **1.4723** | 0.0694 |  | **1.6070** | 0.2017 |
| KIAA1068 | **0.9504** | 0.2011 |  | **1.6040** | 0.2790 |
| EFA6R | **1.1498** | 0.1008 |  | **1.6037** | 0.1415 |
| GPATC2 | **0.9551** | 0.2058 |  | **1.6036** | 0.1763 |
| LOC81558 | **0.9297** | 0.0973 |  | **1.5995** | 0.1816 |
| TMEM49 | **1.3658** | 0.1556 |  | **1.5976** | 0.1240 |
| RAB4B | **1.8202** | 0.0421 |  | **1.5955** | 0.1320 |
| PHC2 | **0.8812** | 0.1770 |  | **1.5953** | 0.2941 |
| LOC339184 | **1.0219** | 0.1211 |  | **1.5937** | 0.1432 |
| LOC222171 | **1.1874** | 0.0485 |  | **1.5923** | 0.0271 |
| C10ORF77 | **1.1057** | 0.0425 |  | **1.5900** | 0.0426 |
| TDE2L | **1.1364** | 0.1050 |  | **1.5889** | 0.0924 |
| CETN1 | **1.5797** | 0.1991 |  | **1.5887** | 0.2001 |
| DNAJC1 | **1.5871** | 0.3247 |  | **1.5841** | 0.1973 |
| LRRN6A | **1.2356** | 0.2877 |  | **1.5828** | 0.1006 |
| LOC344657 | **1.0391** | 0.1611 |  | **1.5812** | 0.3140 |
| CGI-48 | **0.9118** | 0.0837 |  | **1.5795** | 0.0781 |
| TIMM17A | **1.3960** | 0.4365 |  | **1.5783** | 0.2878 |
| MRPL24 | **1.3287** | 0.1415 |  | **1.5779** | 0.2118 |
| KIAA1411 | **0.9107** | 0.1204 |  | **1.5776** | 0.1812 |
| ANKRD10 | **1.1996** | 0.0630 |  | **1.5762** | 0.0692 |
| LOC339745 | **1.3682** | 0.0417 |  | **1.5754** | 0.0224 |
| HTR2C | **1.2671** | 0.1833 |  | **1.5752** | 0.4759 |
| NPTX1 | **1.2719** | 0.1789 |  | **1.5744** | 0.1717 |
| MGC2494 | **0.7550** | 0.1285 |  | **1.5736** | 0.1573 |
| KIAA1305 | **1.3352** | 0.0984 |  | **1.5652** | 0.1383 |
| KLP1 | **0.7558** | 0.1335 |  | **1.5647** | 0.1617 |
| MRPL37 | **0.9974** | 0.0496 |  | **1.5644** | 0.0558 |
| RAMP3 | **1.0539** | 0.2094 |  | **1.5636** | 0.1808 |
| ZNF451 | **0.8885** | 0.0414 |  | **1.5613** | 0.0359 |
| LOC90485 | **0.8749** | 0.1678 |  | **1.5609** | 0.2144 |
| TAS2R9 | **0.9844** | 0.1076 |  | **1.5596** | 0.1515 |
| LYZL6 | **1.0592** | 0.1577 |  | **1.5594** | 0.2384 |
| FLJ12442 | **0.9258** | 0.0714 |  | **1.5575** | 0.0956 |
| LOC317671 | **1.1771** | 0.2833 |  | **1.5544** | 0.3010 |
| MAP7 | **1.1575** | 0.1004 |  | **1.5535** | 0.0787 |
| CABP7 | **1.1132** | 0.1438 |  | **1.5505** | 0.2046 |
| RBP7 | **1.0404** | 0.0378 |  | **1.5485** | 0.0523 |
| FBXW9 | **1.7495** | 0.1520 |  | **1.5468** | 0.1845 |
| SLC7A1 | **1.5191** | 0.1539 |  | **1.5451** | 0.0062 |
| LOC387638 | **0.8643** | 0.1453 |  | **1.5436** | 0.3329 |
| C14ORF126 | **1.0632** | 0.0993 |  | **1.5436** | 0.1314 |
| NS5ATP13TP2 | **0.7569** | 0.0941 |  | **1.5430** | 0.1319 |
| SOX14 | **1.0630** | 0.1828 |  | **1.5412** | 0.1333 |
| FKSG14 | **0.8526** | 0.1439 |  | **1.5405** | 0.1373 |
| MPZL1 | **1.2508** | 0.3650 |  | **1.5402** | 0.3280 |
| TMEM57 | **1.0069** | 0.1786 |  | **1.5400** | 0.2631 |
| DHCR7 | **1.3326** | 0.1746 |  | **1.5395** | 0.1802 |
| KLF7 | **1.0553** | 0.0671 |  | **1.5394** | 0.1328 |
| LOC390999 | **1.1357** | 0.1980 |  | **1.5393** | 0.2258 |
| TRIM69 | **1.1290** | 0.2017 |  | **1.5385** | 0.2945 |
| PBX1 | **1.4839** | 0.0968 |  | **1.5385** | 0.0659 |
| MGC3731 | **1.2464** | 0.1523 |  | **1.5354** | 0.0977 |
| FLJ33641 | **1.0565** | 0.1006 |  | **1.5353** | 0.1886 |
| GDF11 | **1.0207** | 0.0626 |  | **1.5341** | 0.0520 |
| SSX4B | **1.0203** | 0.0603 |  | **1.5323** | 0.0381 |
| FOXN4 | **1.3134** | 0.8130 |  | **1.5320** | 0.7285 |
| HIST4H4 | **1.1453** | 0.1041 |  | **1.5297** | 0.1409 |
| MYBPH | **0.9672** | 0.1483 |  | **1.5284** | 0.1733 |
| ASB6 | **1.5887** | 0.1827 |  | **1.5262** | 0.0545 |
| MGC:13379 | **0.9638** | 0.2090 |  | **1.5249** | 0.1720 |
| IFRD2 | **1.3048** | 0.1974 |  | **1.5231** | 0.2622 |
| TTLL1 | **1.0529** | 0.0359 |  | **1.5225** | 0.0687 |
| C12ORF22 | **0.8197** | 0.1834 |  | **1.5212** | 0.2223 |
| ARHGEF16 | **0.9566** | 0.1767 |  | **1.5211** | 0.2710 |
| ZNF197 | **0.9947** | 0.0902 |  | **1.5201** | 0.0393 |
| ABCA9 | **0.9875** | 0.0978 |  | **1.5186** | 0.1434 |
| GABARAPL1 | **1.0204** | 0.1382 |  | **1.5186** | 0.1092 |
| C7ORF34 | **1.0466** | 0.1280 |  | **1.5185** | 0.1195 |
| MGC24047 | **0.7256** | 0.1273 |  | **1.5172** | 0.1437 |
| DKFZP434B168 | **1.0500** | 0.1662 |  | **1.5137** | 0.1350 |
| FLJ35709 | **0.9333** | 0.1703 |  | **1.5128** | 0.1391 |
| SIAH1 | **1.2686** | 0.1506 |  | **1.5126** | 0.1506 |
| GP2 | **1.2960** | 0.1598 |  | **1.5117** | 0.1191 |
| FAT2 | **1.3726** | 0.1087 |  | **1.5092** | 0.1874 |
| TLL2 | **1.1531** | 0.4359 |  | **1.5071** | 0.3832 |
| FSTL1 | **1.0630** | 0.0871 |  | **1.5066** | 0.2025 |
| SNRPD3 | **1.4294** | 0.0274 |  | **1.5060** | 0.0874 |
| LOC164153 | **1.1411** | 0.0758 |  | **1.5056** | 0.0541 |
| ARNT | **1.2066** | 0.0471 |  | **1.5046** | 0.0910 |
| FLJ20403 | **0.8909** | 0.0890 |  | **1.5003** | 0.1441 |
| C6ORF15 | **0.6772** | 0.0673 |  | **1.4993** | 0.1426 |
| IMP5 | **1.1604** | 0.2071 |  | **1.4981** | 0.3205 |
| FGF20 | **1.1587** | 0.0749 |  | **1.4981** | 0.0989 |
| PRH1 | **1.4048** | 0.3137 |  | **1.4962** | 0.3775 |
| FBLP-1 | **1.2844** | 0.1468 |  | **1.4961** | 0.1992 |
| FOSL2 | **1.0417** | 0.0969 |  | **1.4961** | 0.0352 |
| BMP10 | **1.0025** | 0.0604 |  | **1.4946** | 0.0383 |
| VKORC1 | **1.2685** | 0.0632 |  | **1.4936** | 0.0373 |
| CHST11 | **1.0684** | 0.2420 |  | **1.4921** | 0.1376 |
| FLJ45744 | **1.1325** | 0.0644 |  | **1.4908** | 0.1309 |
| KIAA0676 | **0.6990** | 0.2238 |  | **1.4901** | 0.1736 |
| FREM1 | **1.0612** | 0.2045 |  | **1.4896** | 0.3689 |
| HOXB3 | **0.8733** | 0.1357 |  | **1.4889** | 0.2428 |
| ARC92 | **0.9324** | 0.0099 |  | **1.4885** | 0.0799 |
| LSM6 | **1.3538** | 0.1698 |  | **1.4871** | 0.1408 |
| CCL1 | **1.3745** | 0.1610 |  | **1.4864** | 0.2308 |
| TTF1 | **0.9524** | 0.4521 |  | **1.4844** | 0.5018 |
| DKK1 | **0.9360** | 0.1965 |  | **1.4834** | 0.0983 |
| OR51B5 | **0.8118** | 0.0765 |  | **1.4811** | 0.0528 |
| ZNF259 | **1.3314** | 0.1125 |  | **1.4809** | 0.0782 |
| DHX38 | **1.1324** | 0.1653 |  | **1.4805** | 0.1022 |
| EIF3S6 | **1.4584** | 0.3374 |  | **1.4804** | 0.4420 |
| LOC342933 | **0.7470** | 0.1003 |  | **1.4799** | 0.0937 |
| CARD9 | **1.0411** | 0.0444 |  | **1.4793** | 0.0193 |
| RLN2 | **0.9540** | 0.0899 |  | **1.4789** | 0.0894 |
| PPM1L | **0.9005** | 0.1007 |  | **1.4786** | 0.3259 |
| GPR8 | **1.3263** | 0.1821 |  | **1.4774** | 0.3595 |
| SEPN1 | **1.2598** | 0.2250 |  | **1.4769** | 0.2668 |
| MRPL38 | **1.0802** | 0.3163 |  | **1.4755** | 0.4767 |
| WDR44 | **1.4590** | 0.8129 |  | **1.4750** | 0.6801 |
| LHX6 | **1.5480** | 0.0420 |  | **1.4748** | 0.1051 |
| CNR2 | **1.5419** | 0.0506 |  | **1.4738** | 0.0600 |
| FLJ41993 | **0.8226** | 0.3845 |  | **1.4720** | 0.4593 |
| FMOD | **1.3366** | 0.2010 |  | **1.4716** | 0.2175 |
| LOC146562 | **0.7195** | 0.0505 |  | **1.4705** | 0.0825 |
| SNX8 | **0.9360** | 0.1582 |  | **1.4705** | 0.2215 |
| VAMP2 | **0.9464** | 0.2031 |  | **1.4699** | 0.2809 |
| KIAA0563 | **1.0835** | 0.0421 |  | **1.4698** | 0.1440 |
| OTOR | **0.8096** | 0.3308 |  | **1.4691** | 0.2875 |
| B3GAT3 | **1.1136** | 0.0796 |  | **1.4687** | 0.1480 |
| SLAMF8 | **1.1857** | 0.1584 |  | **1.4672** | 0.2210 |
| C20ORF17 | **1.1664** | 0.2319 |  | **1.4669** | 0.2289 |
| WDR5 | **0.9953** | 0.0819 |  | **1.4666** | 0.1159 |
| BICD2 | **1.2533** | 0.0784 |  | **1.4655** | 0.0791 |
| CIR | **0.8426** | 0.1203 |  | **1.4647** | 0.2507 |
| ZPBP | **1.1053** | 0.2237 |  | **1.4644** | 0.1209 |
| CIDEB | **1.0333** | 0.1422 |  | **1.4640** | 0.3516 |
| SRC | **1.5033** | 0.4477 |  | **1.4632** | 0.4338 |
| FBXL11 | **1.2523** |  |  | **1.4624** |  |
| FMR2 | **1.1234** | 0.1319 |  | **1.4605** | 0.1506 |
| MGC72001 | **0.9087** | 0.0925 |  | **1.4604** | 0.0770 |
| SUSD2 | **0.9894** | 0.0927 |  | **1.4600** | 0.0938 |
| SGTA | **1.2634** |  |  | **1.4595** |  |
| IRF6 | **1.0692** | 0.1342 |  | **1.4589** | 0.1383 |
| GYS1 | **0.9412** | 0.1678 |  | **1.4578** | 0.2482 |
| GSTM1 | **1.1091** | 0.1831 |  | **1.4571** | 0.0968 |
| HSPA14 | **1.1997** | 0.1315 |  | **1.4553** | 0.0512 |
| DNAJC8 | **0.8346** | 0.0357 |  | **1.4548** | 0.0525 |
| CCR9 | **1.1077** | 0.0116 |  | **1.4544** | 0.2328 |
| MBD1 | **0.7302** | 0.2129 |  | **1.4539** | 0.1637 |
| RAP2C | **1.0101** | 0.2172 |  | **1.4537** | 0.3200 |
| GPRC5B | **0.9428** | 0.0813 |  | **1.4534** | 0.0887 |
| MUSK | **0.9583** | 0.1005 |  | **1.4523** | 0.0812 |
| C5ORF6 | **1.0416** | 0.0549 |  | **1.4522** | 0.1184 |
| WARS2 | **1.1366** | 0.1135 |  | **1.4505** | 0.2467 |
| CECR5 | **1.0530** | 0.1521 |  | **1.4453** | 0.1078 |
| ALDH1A1 | **1.1506** | 0.0202 |  | **1.4452** | 0.0860 |
| LOC646603 | **0.9325** | 0.0624 |  | **1.4448** | 0.1502 |
| PTMA | **1.0811** | 0.0678 |  | **1.4442** | 0.1369 |
| HTR3A | **0.9235** | 0.1382 |  | **1.4438** | 0.0887 |
| ZNF584 | **0.8936** | 0.1375 |  | **1.4434** | 0.1372 |
| RASSF3 | **1.1894** | 0.1342 |  | **1.4433** | 0.1979 |
| C8ORF21 | **1.1151** | 0.0596 |  | **1.4428** | 0.0313 |
| LOC340578 | **0.8258** | 0.1851 |  | **1.4427** | 0.2115 |
| PCDHB11 | **1.4199** | 0.0576 |  | **1.4413** | 0.0664 |
| LOC340168 | **0.7085** | 0.0326 |  | **1.4410** | 0.0599 |
| MYO1G | **1.2765** | 0.1314 |  | **1.4407** | 0.1184 |
| DLGAP2 | **1.2805** | 0.1792 |  | **1.4407** | 0.3095 |
| RFPL1 | **1.2714** | 0.1137 |  | **1.4391** | 0.1354 |
| MAGEB2 | **1.0511** | 0.1353 |  | **1.4381** | 0.1547 |
| FLJ22573 | **0.8037** | 0.0788 |  | **1.4380** | 0.1277 |
| ZNF313 | **0.9137** |  |  | **1.4376** |  |
| SLC30A4 | **1.4708** | 0.2011 |  | **1.4374** | 0.0921 |
| IDI2 | **0.8937** | 0.0669 |  | **1.4365** | 0.0192 |
| 384D8-2 | **1.1330** | 0.1302 |  | **1.4362** | 0.0361 |
| SH3BP1 | **0.9027** | 0.1736 |  | **1.4356** | 0.2768 |
| EXT1 | **0.8925** | 0.0370 |  | **1.4351** | 0.0793 |
| SASH1 | **1.1549** | 0.1505 |  | **1.4339** | 0.1434 |
| OR6S1 | **1.1934** | 0.0216 |  | **1.4320** | 0.0165 |
| GRPEL2 | **0.9950** | 0.1847 |  | **1.4319** | 0.3460 |
| CLDN2 | **1.0090** | 0.2016 |  | **1.4317** | 0.1701 |
| PKP1 | **1.1021** | 0.1910 |  | **1.4305** | 0.0972 |
| GCAT | **1.0549** | 0.1484 |  | **1.4295** | 0.1325 |
| PILRA | **1.1701** | 0.0969 |  | **1.4280** | 0.1643 |
| FLJ40722 | **0.9736** | 0.1321 |  | **1.4269** | 0.1772 |
| C18ORF21 | **0.9407** | 0.4524 |  | **1.4266** | 0.5930 |
| NAV2 | **1.2185** | 0.1076 |  | **1.4264** | 0.0394 |
| NOX3 | **0.9657** | 0.2344 |  | **1.4234** | 0.1074 |
| LOC284194 | **0.9569** | 0.0275 |  | **1.4231** | 0.0960 |
| H2BFWT | **1.0131** | 0.1113 |  | **1.4215** | 0.1627 |
| HS3ST3B1 | **0.9622** | 0.2144 |  | **1.4202** | 0.1988 |
| PIWIL3 | **0.8280** | 0.1891 |  | **1.4195** | 0.2405 |
| RDH13 | **0.7416** | 0.0921 |  | **1.4187** | 0.1824 |
| CGREF1 | **0.6602** | 0.0999 |  | **1.4183** | 0.0273 |
| TBCA | **1.4022** | 0.2916 |  | **1.4182** | 0.3979 |
| KRTAP5-5 | **1.4748** | 0.3028 |  | **1.4171** | 0.2049 |
| HOXA5 | 1.0658 | 0.1689 |  | 1.4169 | 0.1771 |
| IQGAP2 | **-0.5982** | 0.0657 |  | **-1.0718** | 0.1437 |
| APRT | **-0.3762** | 0.0400 |  | **-1.0719** | 0.2599 |
| FLJ11078 | **-0.4312** | 0.1129 |  | **-1.0726** | 0.2378 |
| PET112L | **-0.3986** | 0.0935 |  | **-1.0728** | 0.1732 |
| CGB1 | **-0.5265** | 0.0926 |  | **-1.0742** | 0.3202 |
| FLJ22173 | **-0.3740** | 0.0134 |  | **-1.0745** | 0.0953 |
| PKD2L1 | **-0.6296** | 0.0732 |  | **-1.0745** | 0.1115 |
| ARFGEF2 | **-0.5148** | 0.0198 |  | **-1.0748** | 0.0660 |
| GJA8 | **-0.3582** | 0.0826 |  | **-1.0766** | 0.0767 |
| LILRB5 | **-0.5723** | 0.0625 |  | **-1.0773** | 0.1736 |
| AQP8 | **-0.6458** | 0.0814 |  | **-1.0776** | 0.1214 |
| NALP12 | **-0.3561** | 0.0914 |  | **-1.0779** | 0.1996 |
| LOC643418 | **-0.3733** | 0.0357 |  | **-1.0783** | 0.1416 |
| MMP11 | **-0.6156** | 0.0897 |  | **-1.0810** | 0.4826 |
| TAS2R10 | **-0.4207** | 0.0594 |  | **-1.0811** | 0.0782 |
| CTGLF5 | **-0.5688** | 0.1010 |  | **-1.0814** | 0.1882 |
| KIAA1609 | **-0.5759** | 0.0386 |  | **-1.0815** | 0.0251 |
| RLF | **-0.5148** | 0.0477 |  | **-1.0819** | 0.1538 |
| FBXW11 | **-0.6345** | 0.0133 |  | **-1.0819** | 0.0724 |
| TARBP2 | **-0.6295** | 0.0872 |  | **-1.0824** | 0.2521 |
| TRAM2 | **-0.5108** | 0.0711 |  | **-1.0834** | 0.2816 |
| SERPINI1 | **-0.4324** | 0.0790 |  | **-1.0836** | 0.0444 |
| ARSD | **-0.3496** | 0.0709 |  | **-1.0853** | 0.3145 |
| MANEA | **-0.5355** | 0.0187 |  | **-1.0855** | 0.1512 |
| C9ORF115 | **-0.6373** | 0.1322 |  | **-1.0872** | 0.2309 |
| EDNRB | **-0.8056** | 0.0870 |  | **-1.0875** | 0.1993 |
| OR11H12 | **-0.6032** | 0.1044 |  | **-1.0877** | 0.1974 |
| ALX3 | **-0.3822** | 0.0708 |  | **-1.0889** | 0.2214 |
| PMCH | **-0.3614** | 0.0544 |  | **-1.0903** | 0.0601 |
| FLJ40142 | **-0.6397** | 0.0975 |  | **-1.0905** | 0.3947 |
| GPT | **-0.4151** | 0.0859 |  | **-1.0909** | 0.1749 |
| FLJ32112 | **-0.3897** | 0.0346 |  | **-1.0920** | 0.1761 |
| POFUT2 | **-0.3016** | 0.0441 |  | **-1.0921** | 0.3090 |
| MFTC | **-0.6157** | 0.1310 |  | **-1.0922** | 0.2755 |
| KIAA0971 | **-0.4197** | 0.0517 |  | **-1.0933** | 0.0994 |
| ALS2CR3 | **-0.3426** | 0.0504 |  | **-1.0934** | 0.1002 |
| C15ORF24 | **-0.5668** | 0.0531 |  | **-1.0936** | 0.3575 |
| ABLIM2 | **-0.0955** | 0.0786 |  | **-1.0938** | 0.2217 |
| OR2D3 | **-0.4609** | 0.0446 |  | **-1.0949** | 0.1206 |
| FLJ10803 | **-0.4189** | 0.1170 |  | **-1.0953** | 0.2533 |
| PS1D | **-0.3617** | 0.0322 |  | **-1.0956** | 0.1011 |
| IL12RB2 | **-0.3563** | 0.0906 |  | **-1.0961** | 0.2045 |
| SFTPA1 | **-0.5150** | 0.0343 |  | **-1.0971** | 0.1408 |
| LMNA | **-0.6231** | 0.1475 |  | **-1.0976** | 0.2061 |
| CNTNAP5 | **-0.3150** | 0.0533 |  | **-1.0992** | 0.2169 |
| ZNF311 | **-0.5535** | 0.0465 |  | **-1.0994** | 0.0912 |
| RBM10 | **-0.3165** | 0.0555 |  | **-1.0996** | 0.1034 |
| FMO2 | **-0.6429** | 0.1519 |  | **-1.1002** | 0.2191 |
| C14ORF138 | **-0.3236** | 0.0391 |  | **-1.1005** | 0.1311 |
| E2F4 | **-0.4005** | 0.0689 |  | **-1.1019** | 0.1903 |
| CCNB3 | **-0.6070** | 0.0423 |  | **-1.1020** | 0.1874 |
| RAB15 | **-0.3274** | 0.0328 |  | **-1.1022** | 0.0944 |
| ABCC5 | **-0.4399** | 0.0940 |  | **-1.1023** | 0.1233 |
| PTGDR | **-0.7596** | 0.0782 |  | **-1.1025** | 0.3062 |
| MB | **-0.6004** | 0.0123 |  | **-1.1028** | 0.0466 |
| TEX12 | **-0.4416** | 0.0245 |  | **-1.1030** | 0.1761 |
| TRALPUSH | **-0.3830** | 0.0663 |  | **-1.1039** | 0.0618 |
| NME2 | **-0.7265** | 0.0534 |  | **-1.1049** | 0.1131 |
| CITED1 | **-0.6119** | 0.0517 |  | **-1.1060** | 0.2646 |
| NKD2 | **-0.4056** | 0.0156 |  | **-1.1068** | 0.1687 |
| APOL4 | **-0.5907** | 0.0141 |  | **-1.1071** | 0.1033 |
| ZADH1 | **-0.5939** | 0.1329 |  | **-1.1081** | 0.1577 |
| OSTF1 | **-0.5965** | 0.1929 |  | **-1.1082** | 0.4328 |
| IER3 | **-0.6361** | 0.1146 |  | **-1.1082** | 0.2452 |
| CCRK | **-0.7977** | 0.0878 |  | **-1.1088** | 0.1288 |
| CHRNA6 | **-0.6122** | 0.0733 |  | **-1.1089** | 0.1031 |
| RLBP1 | **-0.4985** | 0.2645 |  | **-1.1101** | 0.4638 |
| MUSTN1 | **-0.5445** | 0.0902 |  | **-1.1102** | 0.2066 |
| QSCN6L1 | **-0.6149** | 0.0370 |  | **-1.1104** | 0.3368 |
| EEF2 | **-0.4279** | 0.0531 |  | **-1.1112** | 0.1319 |
| MRPS22 | **-0.6051** | 0.0708 |  | **-1.1116** | 0.1896 |
| RABL2A | **-0.6074** |  |  | **-1.1135** |  |
| MEST | **-0.5811** | 0.0605 |  | **-1.1152** | 0.1821 |
| CAPZA3 | **-0.3717** | 0.0290 |  | **-1.1153** | 0.1367 |
| PARD6A | **-0.3689** | 0.0198 |  | **-1.1156** | 0.1350 |
| FATE1 | **-0.3316** | 0.0029 |  | **-1.1166** | 0.1025 |
| SLC6A9 | **-0.5748** | 0.0080 |  | **-1.1179** | 0.0442 |
| SUV420H2 | **-0.6613** | 0.0143 |  | **-1.1182** | 0.1284 |
| COL17A1 | **-0.3678** | 0.0541 |  | **-1.1188** | 0.1088 |
| CHP | **-0.4803** | 0.0108 |  | **-1.1193** | 0.1316 |
| C1ORF22 | **-0.5632** | 0.1251 |  | **-1.1193** | 0.2209 |
| DJ971N18.2 | **-0.5350** | 0.1802 |  | **-1.1194** | 0.3491 |
| FABP5 | **-0.4103** | 0.0597 |  | **-1.1195** | 0.1672 |
| GPRC6A | **-0.8695** | 0.0960 |  | **-1.1197** | 0.3217 |
| PPP2R2A | **-0.6684** | 0.0487 |  | **-1.1198** | 0.3379 |
| YOD1 | **-0.6678** | 0.0984 |  | **-1.1203** | 0.2804 |
| LOC285636 | **-0.3569** | 0.0392 |  | **-1.1206** | 0.1529 |
| OR9G1 | **-0.5039** | 0.0424 |  | **-1.1211** | 0.2156 |
| MGC39497 | **-0.4457** | 0.0214 |  | **-1.1218** | 0.0602 |
| TTC10 | **-0.3890** | 0.0146 |  | **-1.1220** | 0.0982 |
| TP53BP2 | **-0.5592** | 0.0777 |  | **-1.1221** | 0.0668 |
| THAP1 | **-0.6210** | 0.1164 |  | **-1.1227** | 0.3504 |
| NXPH1 | **-0.3981** | 0.0490 |  | **-1.1230** | 0.1104 |
| C19ORF7 | **-0.5695** | 0.0284 |  | **-1.1231** | 0.2958 |
| SPG3A | **-0.4485** | 0.1240 |  | **-1.1267** | 0.1978 |
| MUC17 | **-0.5092** | 0.0765 |  | **-1.1292** | 0.0981 |
| RP11-56A21.1 | **-0.5642** | 0.1313 |  | **-1.1297** | 0.3603 |
| HSPH1 | **-0.3771** | 0.0521 |  | **-1.1330** | 0.0355 |
| ZFD25 | **-0.4148** | 0.0256 |  | **-1.1331** | 0.1671 |
| RING1 | **-0.5838** | 0.0286 |  | **-1.1335** | 0.0996 |
| OR2T12 | **-0.6371** | 0.0699 |  | **-1.1337** | 0.2055 |
| SHOX2 | **-0.6034** | 0.0950 |  | **-1.1337** | 0.0510 |
| B3GNT1 | **-0.3748** | 0.0440 |  | **-1.1343** | 0.0981 |
| FLJ21308 | **-0.3426** | 0.0739 |  | **-1.1344** | 0.0576 |
| C10ORF30 | **-0.5343** | 0.0289 |  | **-1.1346** | 0.1540 |
| PRRG3 | **-0.3988** | 0.0572 |  | **-1.1351** | 0.0918 |
| DKFZP564G2022 | **-0.4371** | 0.0830 |  | **-1.1370** | 0.2058 |
| VIT | **-0.3363** | 0.0147 |  | **-1.1370** | 0.2301 |
| LIMD1 | **-0.5352** | 0.0373 |  | **-1.1384** | 0.2071 |
| ASTL | **-0.3732** | 0.1185 |  | **-1.1385** | 0.2223 |
| PARP1 | **-0.4083** | 0.0365 |  | **-1.1386** | 0.2480 |
| DKFZP564O243 | **-0.4537** | 0.0934 |  | **-1.1408** | 0.2208 |
| FLJ46675 | **-0.3942** | 0.1072 |  | **-1.1413** | 0.3186 |
| LOC389257 | **-0.3505** | 0.0088 |  | **-1.1417** | 0.1353 |
| GDF10 | **-0.6058** | 0.1621 |  | **-1.1431** | 0.3554 |
| EBAG9 | **-0.6249** | 0.0447 |  | **-1.1431** | 0.1724 |
| JMJD1A | **-0.5266** | 0.0551 |  | **-1.1433** | 0.0284 |
| VEZATIN | **-0.4441** | 0.0859 |  | **-1.1443** | 0.1783 |
| SENP7 | **-0.6253** | 0.0443 |  | **-1.1446** | 0.1118 |
| SVIL | **-0.4032** | 0.0620 |  | **-1.1449** | 0.1806 |
| PHYHIP | **-0.6281** | 0.0516 |  | **-1.1462** | 0.1781 |
| SEMA3A | **-0.3757** | 0.0293 |  | **-1.1464** | 0.0466 |
| HEMGN | **-0.3207** | 0.0835 |  | **-1.1478** | 0.1595 |
| RGMB | **-0.3993** | 0.0311 |  | **-1.1488** | 0.0249 |
| RKHD1 | **-0.6828** | 0.0280 |  | **-1.1492** | 0.1441 |
| XRCC3 | **-0.6153** | 0.1194 |  | **-1.1502** | 0.1355 |
| PLCB4 | **-0.3645** | 0.0810 |  | **-1.1514** | 0.3461 |
| ZSWIM2 | **-0.6292** | 0.0811 |  | **-1.1518** | 0.3046 |
| SCUBE2 | **-0.5324** | 0.0212 |  | **-1.1518** | 0.0485 |
| H2AFZ | **-0.5928** | 0.0507 |  | **-1.1524** | 0.2178 |
| OR5K4 | **-0.2948** | 0.0599 |  | **-1.1530** | 0.2049 |
| SIVA | **-0.5775** | 0.1526 |  | **-1.1532** | 0.3920 |
| KIAA1468 | **-0.5181** | 0.0486 |  | **-1.1532** | 0.1158 |
| C5ORF4 | **-0.3476** | 0.0046 |  | **-1.1533** | 0.1706 |
| GRLF1 | **-0.4237** | 0.0312 |  | **-1.1539** | 0.1485 |
| TCN2 | **-0.6332** | 0.0882 |  | **-1.1549** | 0.0996 |
| CEBPE | **-0.6562** | 0.1205 |  | **-1.1552** | 0.1884 |
| ASF1A | **-0.5894** | 0.0799 |  | **-1.1563** | 0.1286 |
| TINP1 | **-0.5423** | 0.0818 |  | **-1.1571** | 0.3905 |
| IRX1 | **-0.5123** | 0.0291 |  | **-1.1574** | 0.1375 |
| R30953_1 | **-0.4079** | 0.0247 |  | **-1.1574** | 0.0197 |
| VNN3 | **-0.3775** | 0.0587 |  | **-1.1596** | 0.0740 |
| TAS2R5 | **-0.5854** | 0.0463 |  | **-1.1597** | 0.0486 |
| IFI30 | **-0.3804** | 0.0378 |  | **-1.1620** | 0.1257 |
| GFRA3 | **-0.7037** | 0.1104 |  | **-1.1679** | 0.2697 |
| AFM | **-0.4482** | 0.0481 |  | **-1.1695** | 0.2328 |
| HOXB13 | **-0.3300** | 0.0878 |  | **-1.1704** | 0.1815 |
| LYZL2 | **-0.3874** | 0.0545 |  | **-1.1706** | 0.1526 |
| HDHD4 | **-0.3948** | 0.0638 |  | **-1.1711** | 0.1480 |
| FLJ10006 | **-0.3863** | 0.0946 |  | **-1.1712** | 0.1018 |
| FRMPD1 | **-0.4041** | 0.0276 |  | **-1.1716** | 0.1534 |
| SOD2 | **-0.5815** | 0.0252 |  | **-1.1723** | 0.1142 |
| MKKS | **-0.6187** | 0.0647 |  | **-1.1737** | 0.2255 |
| C10ORF91 | **-0.3848** | 0.0693 |  | **-1.1737** | 0.4440 |
| TUBD1 | **-0.4052** | 0.0677 |  | **-1.1760** | 0.2359 |
| FOLR2 | **-0.6157** |  |  | **-1.1762** |  |
| KIAA0555 | **-0.4703** | 0.0849 |  | **-1.1764** | 0.1762 |
| SYCP2 | **-0.3826** | 0.0316 |  | **-1.1771** | 0.1629 |
| POLE3 | **-0.5613** | 0.0235 |  | **-1.1777** | 0.1485 |
| GULP1 | **-0.5859** | 0.0587 |  | **-1.1784** | 0.1189 |
| OR52D1 | **-0.5095** | 0.0197 |  | **-1.1793** | 0.1092 |
| OR2AE1 | **-0.5750** | 0.0502 |  | **-1.1795** | 0.0663 |
| C20ORF13 | **-0.5638** | 0.0182 |  | **-1.1796** | 0.1045 |
| MAK3 | **-0.6914** | 0.0808 |  | **-1.1809** | 0.1980 |
| PHOSPHO2 | **-0.4465** | 0.0372 |  | **-1.1816** | 0.1779 |
| LSM11 | **-0.5262** | 0.0411 |  | **-1.1835** | 0.1488 |
| C14ORF127 | **-0.4162** | 0.0479 |  | **-1.1844** | 0.0610 |
| BACH1 | **-0.3959** | 0.0752 |  | **-1.1844** | 0.1900 |
| CLDN6 | **-0.3400** | 0.0832 |  | **-1.1846** | 0.2511 |
| KLF15 | **-0.6402** | 0.0869 |  | **-1.1849** | 0.1365 |
| LOC162993 | **-0.4712** | 0.0471 |  | **-1.1850** | 0.1503 |
| ASAM | **-0.6046** | 0.0168 |  | **-1.1851** | 0.0766 |
| TMOD4 | **-0.5787** | 0.0808 |  | **-1.1852** | 0.2627 |
| SKP1A | **-0.3698** | 0.0303 |  | **-1.1857** | 0.0374 |
| BTN2A1 | **-0.3288** | 0.0713 |  | **-1.1858** | 0.0703 |
| ZRANB1 | **-0.3814** | 0.0953 |  | **-1.1875** | 0.2489 |
| PDE6G | **-0.6100** | 0.0793 |  | **-1.1881** | 0.2660 |
| LOC389834 | **-0.6445** | 0.0932 |  | **-1.1881** | 0.3196 |
| UNC5D | **-0.3416** | 0.0988 |  | **-1.1890** | 0.0764 |
| ATP5G2 | **-0.6499** | 0.0779 |  | **-1.1892** | 0.2009 |
| TSPAN5 | **-0.5139** | 0.0402 |  | **-1.1896** | 0.3016 |
| CISH | **-0.6810** | 0.0120 |  | **-1.1903** | 0.1450 |
| ANGPTL4 | **-0.5898** | 0.0615 |  | **-1.1908** | 0.1174 |
| RAB2 | **-0.6116** | 0.0904 |  | **-1.1950** | 0.2377 |
| DJ328E19.C1.1 | **-0.3874** | 0.0298 |  | **-1.1950** | 0.0420 |
| H2AFJ | **-0.5590** | 0.0277 |  | **-1.1956** | 0.3639 |
| SLC23A1 | **-0.4317** | 0.0688 |  | **-1.1966** | 0.1269 |
| IGFBP2 | **-0.3559** | 0.0669 |  | **-1.1992** | 0.1418 |
| UBQLN1 | **-0.5250** | 0.0123 |  | **-1.2017** | 0.3400 |
| PEX5 | **-0.3798** | 0.0531 |  | **-1.2022** | 0.1322 |
| ICA1 | **-0.3702** | 0.0395 |  | **-1.2030** | 0.0605 |
| PAX2 | **-0.3810** | 0.1045 |  | **-1.2043** | 0.1988 |
| ZNF225 | **-0.6156** | 0.0199 |  | **-1.2053** | 0.1162 |
| TBC1D17 | **-0.6278** | 0.0404 |  | **-1.2061** | 0.1076 |
| MEPE | **-0.5686** | 0.0353 |  | **-1.2078** | 0.1349 |
| UBE2T | **-0.6645** | 0.0571 |  | **-1.2084** | 0.1568 |
| KIAA1536 | **-0.4389** | 0.0366 |  | **-1.2099** | 0.0605 |
| HRBL | **-0.3415** | 0.0532 |  | **-1.2100** | 0.1621 |
| HCA112 | **-0.3824** | 0.0295 |  | **-1.2120** | 0.0832 |
| BFSP1 | **-0.5241** | 0.0777 |  | **-1.2144** | 0.2670 |
| GYS2 | **-0.4563** | 0.0905 |  | **-1.2147** | 0.1182 |
| NAT6 | **-0.3631** | 0.0739 |  | **-1.2148** | 0.1350 |
| RAB33B | **-0.4663** | 0.0958 |  | **-1.2159** | 0.1825 |
| H3F3A | **-0.5457** | 0.0721 |  | **-1.2173** | 0.0926 |
| MIA2 | **-0.3978** | 0.0866 |  | **-1.2176** | 0.1490 |
| LRRC4 | **-0.5687** | 0.0454 |  | **-1.2178** | 0.2123 |
| TOM1L2 | **-0.3758** | 0.0307 |  | **-1.2178** | 0.1597 |
| GIF | **-0.4578** | 0.0501 |  | **-1.2199** | 0.1580 |
| XKRY2 | **-0.3816** | 0.0372 |  | **-1.2199** | 0.1651 |
| CABP1 | **-0.3856** | 0.0654 |  | **-1.2202** | 0.1516 |
| CHD2 | **-0.5992** | 0.1099 |  | **-1.2203** | 0.1709 |
| NR0B2 | **-0.5977** |  |  | **-1.2215** |  |
| SSFA2 | **-0.5268** | 0.0908 |  | **-1.2225** | 0.2260 |
| ITGB1 | **-0.5853** | 0.1268 |  | **-1.2234** | 0.2334 |
| NLN | **-0.6851** |  |  | **-1.2249** |  |
| FLJ33360 | **-0.5611** | 0.0620 |  | **-1.2252** | 0.2154 |
| GYG2 | **-0.4080** | 0.0533 |  | **-1.2257** | 0.1228 |
| NR3C1 | **-0.6296** | 0.1297 |  | **-1.2260** | 0.2469 |
| BAI3 | **-0.7954** | 0.1152 |  | **-1.2263** | 0.1272 |
| OR51V1 | **-0.3799** | 0.0605 |  | **-1.2269** | 0.1456 |
| DIAPH3 | **-0.4141** | 0.0781 |  | **-1.2277** | 0.2241 |
| ARHGEF1 | **-0.5235** | 0.0557 |  | **-1.2278** | 0.1025 |
| APOC1 | **-0.6340** | 0.0920 |  | **-1.2323** | 0.0733 |
| NOL4 | **-0.5838** | 0.1196 |  | **-1.2325** | 0.3866 |
| CYP3A4 | **-0.2759** | 0.0224 |  | **-1.2336** | 0.0055 |
| IL13RA1 | **-0.3376** | 0.0446 |  | **-1.2338** | 0.1648 |
| ALG10 | **-0.4002** | 0.0229 |  | **-1.2347** | 0.2078 |
| BAT1 | **-0.3908** | 0.0674 |  | **-1.2357** | 0.0694 |
| PRDM1 | **-0.4355** | 0.0707 |  | **-1.2358** | 0.1434 |
| RPH3AL | **-0.5754** | 0.0955 |  | **-1.2390** | 0.2248 |
| CLDN11 | **-0.3995** | 0.0464 |  | **-1.2414** | 0.0861 |
| ATP6V0D2 | **-0.4752** | 0.0808 |  | **-1.2420** | 0.2704 |
| C18ORF19 | **-0.6681** | 0.0328 |  | **-1.2439** | 0.2345 |
| OR8J3 | **-0.4171** | 0.0658 |  | **-1.2445** | 0.1884 |
| PDXP | **-0.6191** | 0.0889 |  | **-1.2446** | 0.1491 |
| D4S234E | **-0.3891** | 0.0300 |  | **-1.2455** | 0.0980 |
| C9ORF85 | **-0.4938** | 0.0634 |  | **-1.2477** | 0.2799 |
| FAM128A | **-0.3959** | 0.0452 |  | **-1.2486** | 0.1180 |
| POTE8 | **-0.3811** | 0.0502 |  | **-1.2498** | 0.1363 |
| LOC284402 | **-0.5354** | 0.0340 |  | **-1.2548** | 0.2294 |
| WNT5B | **-0.5636** | 0.0195 |  | **-1.2567** | 0.1586 |
| FHL5 | **-0.4233** | 0.0523 |  | **-1.2567** | 0.0064 |
| SENP6 | **-0.6688** | 0.0725 |  | **-1.2581** | 0.2610 |
| LRRC51 | **-0.3874** | 0.0106 |  | **-1.2583** | 0.1762 |
| CLMN | **-0.4163** | 0.0523 |  | **-1.2590** | 0.1156 |
| ZNF232 | **-0.5563** | 0.0229 |  | **-1.2606** | 0.0546 |
| CSGLCA-T | **-0.5343** | 0.0309 |  | **-1.2619** | 0.1386 |
| PITPNM3 | **-0.5984** | 0.0139 |  | **-1.2626** | 0.1494 |
| FARSLB | **-0.4314** | 0.0379 |  | **-1.2643** | 0.2106 |
| SSX2IP | **-0.3407** | 0.0482 |  | **-1.2644** | 0.2581 |
| RAB34 | **-0.4098** | 0.0663 |  | **-1.2645** | 0.1074 |
| PUNC | **-0.5894** | 0.0713 |  | **-1.2654** | 0.1093 |
| OR2A14 | **-0.4212** | 0.0402 |  | **-1.2681** | 0.1201 |
| CLN3 | **-0.3681** | 0.0452 |  | **-1.2698** | 0.3086 |
| CCL11 | **-0.5623** | 0.0647 |  | **-1.2701** | 0.1663 |
| DLD | **-0.4279** | 0.0388 |  | **-1.2702** | 0.0289 |
| ABCD1 | **-0.5952** |  |  | **-1.2708** |  |
| C10ORF46 | **-0.6442** | 0.0428 |  | **-1.2708** | 0.2173 |
| AP3S1 | **-0.6328** | 0.0454 |  | **-1.2712** | 0.2166 |
| DHX32 | **-0.6036** | 0.0560 |  | **-1.2715** | 0.0909 |
| MGC57211 | **-0.4449** | 0.0239 |  | **-1.2716** | 0.0826 |
| FLJ23834 | **-0.3703** | 0.0250 |  | **-1.2723** | 0.0767 |
| SALL2 | **-0.3613** | 0.0447 |  | **-1.2727** | 0.0705 |
| EPHA10 | **-0.8404** | 0.0204 |  | **-1.2728** | 0.1368 |
| KIAA0319L | **-0.3456** | 0.0538 |  | **-1.2734** | 0.0916 |
| THOC1 | **-0.6012** | 0.0513 |  | **-1.2742** | 0.0911 |
| GMEB1 | **-0.4057** | 0.0329 |  | **-1.2742** | 0.1565 |
| COPEB | **-0.3838** | 0.0727 |  | **-1.2749** | 0.2108 |
| SNX6 | **-0.6363** | 0.0754 |  | **-1.2751** | 0.2138 |
| ACTRT1 | **-0.5563** | 0.0392 |  | **-1.2752** | 0.1494 |
| MYL6 | **-0.6289** | 0.1054 |  | **-1.2764** | 0.1476 |
| PCDHGA3 | **-0.5761** | 0.0327 |  | **-1.2765** | 0.1297 |
| DMP1 | **-0.4136** | 0.0080 |  | **-1.2785** | 0.1335 |
| CTGLF3 | **-0.4602** | 0.1175 |  | **-1.2788** | 0.0915 |
| SLC9A11 | **-0.3658** | 0.0754 |  | **-1.2804** | 0.1572 |
| TGM4 | **-0.6343** | 0.1584 |  | **-1.2817** | 0.2379 |
| ABCB1 | **-0.3096** | 0.0750 |  | **-1.2843** | 0.0957 |
| CUL2 | **-0.7055** | 0.0930 |  | **-1.2845** | 0.2547 |
| TARP | **-0.3487** | 0.0390 |  | **-1.2845** | 0.0977 |
| KIAA1683 | **-0.6474** | 0.0646 |  | **-1.2852** | 0.0740 |
| CBL | **-0.6749** | 0.0249 |  | **-1.2880** | 0.1084 |
| HTR3E | **-0.6388** | 0.0823 |  | **-1.2885** | 0.0757 |
| ACADSB | **-0.4076** | 0.0510 |  | **-1.2893** | 0.0431 |
| MKRN1 | **-0.6036** | 0.0619 |  | **-1.2905** | 0.1760 |
| FBXO36 | **-0.6418** |  |  | **-1.2912** |  |
| SLC3A1 | **-0.4083** | 0.0638 |  | **-1.2916** | 0.1234 |
| ERG | **-0.4094** | 0.0489 |  | **-1.2918** | 0.0718 |
| CD48 | **-0.6470** | 0.1042 |  | **-1.2929** | 0.4526 |
| MARS | **-0.3755** | 0.1094 |  | **-1.2933** | 0.2143 |
| APBA3 | **-0.6514** | 0.1000 |  | **-1.2937** | 0.2034 |
| FLJ39743 | **-0.4095** | 0.0195 |  | **-1.2938** | 0.1435 |
| TBC1D15 | **-0.4175** | 0.0807 |  | **-1.2970** | 0.2134 |
| C20ORF121 | **-0.5525** | 0.0090 |  | **-1.2981** | 0.1175 |
| KIAA0980 | **-0.4197** | 0.0249 |  | **-1.2998** | 0.1198 |
| CYBA | **-0.6896** | 0.0609 |  | **-1.3012** | 0.0699 |
| OPN1MW2 | **-0.6782** | 0.1260 |  | **-1.3029** | 0.4064 |
| C10ORF4 | **-0.5667** | 0.0478 |  | **-1.3106** | 0.2047 |
| CDH10 | **-0.5797** | 0.0483 |  | **-1.3107** | 0.1101 |
| SESN1 | **-0.4170** | 0.0213 |  | **-1.3131** | 0.0686 |
| MYST3 | **-0.6355** | 0.0801 |  | **-1.3149** | 0.1026 |
| PLD2 | **-0.6572** | 0.1129 |  | **-1.3151** | 0.0904 |
| FLJ35767 | **-0.6630** | 0.1525 |  | **-1.3162** | 0.4208 |
| CHGN | **-0.6450** | 0.0619 |  | **-1.3175** | 0.1676 |
| PLGL | **-0.6949** | 0.0308 |  | **-1.3180** | 0.2877 |
| SIX4 | **-0.4118** | 0.0994 |  | **-1.3203** | 0.1633 |
| TNT | **-0.4097** | 0.0920 |  | **-1.3244** | 0.4041 |
| MRPL35 | **-0.4179** | 0.0706 |  | **-1.3277** | 0.3659 |
| HGD | **-0.6763** | 0.0941 |  | **-1.3308** | 0.0706 |
| TMEM91 | **-0.3788** | 0.0158 |  | **-1.3318** | 0.1087 |
| C21ORF63 | **-0.6001** | 0.0795 |  | **-1.3351** | 0.1666 |
| IGFBPL1 | **-0.3247** | 0.1237 |  | **-1.3351** | 0.4006 |
| LYG2 | **-0.4170** | 0.0776 |  | **-1.3354** | 0.3555 |
| MYH6 | **-0.6955** | 0.0813 |  | **-1.3369** | 0.0773 |
| PLA2G7 | **-0.6538** | 0.0817 |  | **-1.3376** | 0.1473 |
| GPR1 | **-0.8524** | 0.1633 |  | **-1.3381** | 0.3340 |
| PPP1R2 | **-0.7513** | 0.0718 |  | **-1.3389** | 0.1003 |
| SPRR1B | **-0.5564** | 0.0383 |  | **-1.3402** | 0.1185 |
| GRIA3 | **-0.6872** | 0.0244 |  | **-1.3407** | 0.0701 |
| ENSA | **-0.3950** | 0.0653 |  | **-1.3419** | 0.1210 |
| SLC27A2 | **-0.6626** | 0.0904 |  | **-1.3433** | 0.1092 |
| C4ORF8 | **-0.6792** | 0.0884 |  | **-1.3437** | 0.2515 |
| LRCH3 | **-0.5239** | 0.0861 |  | **-1.3457** | 0.1934 |
| CRYBB3 | **-0.3786** | 0.0295 |  | **-1.3482** | 0.2283 |
| YWHAQ | **-0.6887** | 0.0679 |  | **-1.3491** | 0.0947 |
| PCMT1 | **-0.4798** | 0.0886 |  | **-1.3500** | 0.0346 |
| GDF8 | **-0.3876** | 0.0727 |  | **-1.3517** | 0.3338 |
| PDLIM1 | **-0.6228** | 0.1339 |  | **-1.3535** | 0.0865 |
| SEC23A | **-0.4018** | 0.0377 |  | **-1.3558** | 0.0419 |
| CLTCL1 | **-0.6154** | 0.0653 |  | **-1.3601** | 0.2382 |
| CSAG1 | **-0.6536** | 0.0485 |  | **-1.3602** | 0.2517 |
| ELOVL3 | **-0.4006** | 0.0531 |  | **-1.3617** | 0.0755 |
| EMP2 | **-0.6760** | 0.1320 |  | **-1.3646** | 0.3692 |
| AFG3L2 | **-0.6531** |  |  | **-1.3674** |  |
| LOC388931 | **-0.4214** | 0.0283 |  | **-1.3692** | 0.1682 |
| BST1 | **-0.6230** | 0.0713 |  | **-1.3703** | 0.1185 |
| OR8B2 | **-0.6367** | 0.0222 |  | **-1.3708** | 0.1241 |
| STRN | **-0.6568** | 0.0686 |  | **-1.3714** | 0.2524 |
| LOC253827 | **-0.4208** | 0.0604 |  | **-1.3739** | 0.1025 |
| SLC35D2 | **-0.4018** | 0.0097 |  | **-1.3803** | 0.1243 |
| LOC200933 | **-0.6673** | 0.0218 |  | **-1.3807** | 0.0835 |
| PRDM2 | **-0.5955** | 0.0483 |  | **-1.3869** | 0.2203 |
| RABL2B | **-0.4634** | 0.0672 |  | **-1.3876** | 0.1198 |
| KLF2 | **-0.6620** | 0.1187 |  | **-1.3897** | 0.3178 |
| ZNF33A | **-0.4271** | 0.0616 |  | **-1.4008** | 0.1984 |
| ZNF236 | **-0.6856** | 0.0421 |  | **-1.4043** | 0.1579 |
| DIAPH2 | **-0.7030** | 0.0550 |  | **-1.4047** | 0.1970 |
| THAP11 | **-0.6901** | 0.0270 |  | **-1.4089** | 0.2159 |
| VDRIP | **-0.6811** | 0.0763 |  | **-1.4130** | 0.2088 |
| EPC2 | **-0.5246** | 0.0619 |  | **-1.4173** | 0.4294 |
| RYBP | **-0.4541** | 0.0815 |  | **-1.4176** | 0.1029 |
| CDH19 | **-0.7171** | 0.0429 |  | **-1.4177** | 0.1923 |
| SLC39A7 | **-0.5766** | 0.0624 |  | **-1.4206** | 0.0487 |
| LYZL1 | **-0.4702** | 0.0819 |  | **-1.4207** | 0.1098 |
| ZNF555 | **-0.6251** | 0.0698 |  | **-1.4209** | 0.2734 |
| ZNF382 | **-0.6052** | 0.0549 |  | **-1.4218** | 0.0820 |
| EPHX2 | **-0.4556** | 0.0780 |  | **-1.4235** | 0.3008 |
| MRO | **-0.4148** | 0.0268 |  | **-1.4268** | 0.1219 |
| EGFL11 | **-0.4182** | 0.0448 |  | **-1.4274** | 0.0921 |
| THAP7 | **-0.4133** | 0.0443 |  | **-1.4282** | 0.2032 |
| DKFZP434C131 | **-0.8218** | 0.0672 |  | **-1.4298** | 0.1563 |
| SLC22A13 | **-0.5391** | 0.0251 |  | **-1.4310** | 0.1831 |
| HS6ST2 | **-0.4188** | 0.0291 |  | **-1.4340** | 0.1174 |
| TMEM16F | **-0.5728** | 0.1156 |  | **-1.4362** | 0.3601 |
| TACR3 | **-0.8583** | 0.1304 |  | **-1.4437** | 0.1701 |
| GPC2 | **-0.6795** | 0.0947 |  | **-1.4450** | 0.3238 |
| MGC24975 | **-0.4218** | 0.0412 |  | **-1.4457** | 0.2508 |
| KIAA0931 | **-0.6318** | 0.0408 |  | **-1.4472** | 0.1159 |
| OSTBETA | **-0.6254** | 0.0527 |  | **-1.4512** | 0.0738 |
| GALNACT-2 | **-0.6292** | 0.0418 |  | **-1.4588** | 0.1471 |
| DKFZP564N2472 | **-0.4580** | 0.0505 |  | **-1.4600** | 0.1750 |
| PXN | **-0.6746** | 0.0714 |  | **-1.4619** | 0.1919 |
| TRAF3IP1 | **-0.5791** | 0.0435 |  | **-1.4627** | 0.2067 |
| PCDHGA4 | **-0.4191** | 0.0259 |  | **-1.4695** | 0.1170 |
| GRIA1 | **-0.7378** | 0.0511 |  | **-1.4700** | 0.1106 |
| LOC51035 | **-0.6565** | 0.0358 |  | **-1.4712** | 0.2175 |
| TIGD2 | **-0.6863** | 0.0514 |  | **-1.4744** | 0.2086 |
| WBP5 | **-0.6789** | 0.0626 |  | **-1.4767** | 0.3129 |
| C14ORF153 | **-0.6903** | 0.0736 |  | **-1.4801** | 0.1663 |
| ZNF615 | **-0.6040** | 0.0379 |  | **-1.4810** | 0.0608 |
| OLFM1 | **-0.7240** | 0.1004 |  | **-1.4815** | 0.2630 |
| PCDHB5 | **-0.4157** | 0.0362 |  | **-1.4896** | 0.1715 |
| GNA12 | **-0.6773** | 0.0973 |  | **-1.4959** | 0.3516 |
| KIAA1361 | **-0.8505** | 0.0444 |  | **-1.5057** | 0.1019 |
| FAM110C | **-0.7358** | 0.0338 |  | **-1.5125** | 0.0669 |
| CPT1C | **-0.4386** | 0.0444 |  | **-1.5134** | 0.1914 |
| KLHL1 | **-0.5957** | 0.0620 |  | **-1.5163** | 0.2538 |
| NAV1 | **-0.4321** | 0.0533 |  | **-1.5229** | 0.1227 |
| TBN | **-0.6434** | 0.0372 |  | **-1.5247** | 0.1493 |
| ENPP5 | **-0.4403** | 0.0737 |  | **-1.5289** | 0.1759 |
| KIR3DL1 | **-0.7006** | 0.0647 |  | **-1.5319** | 0.2009 |
| DDX31 | **-0.4554** | 0.0869 |  | **-1.5328** | 0.1411 |
| ZNF439 | **-0.6335** | 0.0442 |  | **-1.5411** | 0.1660 |
| LRFN3 | **-0.4322** | 0.0336 |  | **-1.5540** | 0.0817 |
| EHMT2 | **-0.4677** | 0.1047 |  | **-1.5550** | 0.2215 |
| LSS | **-0.4704** | 0.0359 |  | **-1.5566** | 0.2133 |
| FTO | **-0.4186** | 0.0403 |  | **-1.5588** | 0.0851 |
| C9 | **-0.4077** | 0.0532 |  | **-1.5704** | 0.2374 |
| CNIH3 | **-0.5679** | 0.0456 |  | **-1.5705** | 0.0517 |
| LOC91431 | **-0.5279** | 0.0662 |  | **-1.5733** | 0.1394 |
| IDH1 | **-0.6188** | 0.0663 |  | **-1.5831** | 0.0216 |
| SEL1L | **-0.5817** | 0.0834 |  | **-1.5949** | 0.2049 |
| TNFRSF11A | **-0.4630** | 0.0662 |  | **-1.5956** | 0.1310 |
| LOC201164 | **-0.6278** | 0.0367 |  | **-1.6139** | 0.1290 |
| HRAS | **-0.4526** | 0.0939 |  | **-1.6172** | 0.0639 |
| APLP2 | **-0.7690** | 0.1121 |  | **-1.6320** | 0.1487 |
| ZNF605 | **-0.4947** | 0.0335 |  | **-1.6367** | 0.0883 |
| DSG1 | **-0.4358** | 0.0504 |  | **-1.6585** | 0.2205 |
| GNAS | **-0.4936** | 0.0634 |  | **-1.6596** | 0.0162 |
| RASA1 | **-0.7209** | 0.0838 |  | **-1.6862** | 0.2715 |
| GORASP2 | **-0.5278** | 0.0112 |  | **-1.6901** | 0.1190 |
| APP | **-0.5073** | 0.0373 |  | **-1.7042** | 0.0963 |
| OLR1 | **-0.7294** | 0.0740 |  | **-1.7418** | 0.1710 |
| HCG27 | **-0.4350** | 0.0541 |  | **-1.7482** | 0.1103 |
| BCL2 | **-0.6812** | 0.0411 |  | **-1.7733** | 0.2029 |
| ACBD5 | **-0.6472** | 0.0617 |  | **-1.7883** | 0.0678 |
| MGC15523 | **-0.5016** | 0.0414 |  | **-1.8567** | 0.0562 |
